# Multi-omics intervention in *Setaria* to dissect climate-resilient traits: Progress and prospects

**DOI:** 10.3389/fpls.2022.892736

**Published:** 2022-08-31

**Authors:** Pooja Rani Aggarwal, Lydia Pramitha, Pooja Choudhary, Roshan Kumar Singh, Pooja Shukla, Manoj Prasad, Mehanathan Muthamilarasan

**Affiliations:** ^1^Department of Plant Sciences, School of Life Sciences, University of Hyderabad, Hyderabad, Telangana, India; ^2^School of Agriculture and Biosciences, Karunya Institute of Technology and Sciences, Coimbatore, Tamil Nadu, India; ^3^National Institute of Plant Genome Research (NIPGR), New Delhi, India

**Keywords:** foxtail millet, climate resilience, C_4_ photosynthesis, nutrition, stress tolerance, integrated omics, breeding

## Abstract

Millets constitute a significant proportion of underutilized grasses and are well known for their climate resilience as well as excellent nutritional profiles. Among millets, foxtail millet (*Setaria italica*) and its wild relative green foxtail (*S. viridis*) are collectively regarded as models for studying broad-spectrum traits, including abiotic stress tolerance, C_4_ photosynthesis, biofuel, and nutritional traits. Since the genome sequence release, the crop has seen an exponential increase in omics studies to dissect agronomic, nutritional, biofuel, and climate-resilience traits. These studies have provided first-hand information on the structure, organization, evolution, and expression of several genes; however, knowledge of the precise roles of such genes and their products remains elusive. Several open-access databases have also been instituted to enable advanced scientific research on these important crops. In this context, the current review enumerates the contemporary trend of research on understanding the climate resilience and other essential traits in *Setaria*, the knowledge gap, and how the information could be translated for the crop improvement of related millets, biofuel crops, and cereals. Also, the review provides a roadmap for studying other underutilized crop species using *Setaria* as a model.

## Introduction

Crop productivity, limited by changing climatic conditions such as increasing temperature, drought, soil salinity, etc., imposes a severe threat on food security worldwide. Evidence suggests that expanding our study and utilizing neglected and underutilized cereals for sustainable agricultural production is imperative. Millets collectively form a group of small-grained cereals, including several distantly related species. This group of crops is cultivated in adverse climatic conditions, such as high temperature and drought, with low agricultural inputs. The most widely grown small millets are finger millet (*Eleusine coracana*), pearl millet (*Pennisetum glaucum*), proso millet (*Panicum miliaceum*), foxtail millet (*Setaria italica*), and barnyard millet (*Echinochloa crus-galli*). Although less popular than major cereal crops, millets are a rich source of protein, resistant starch, micronutrients, antioxidants, and bioactive compounds (Kumar et al., 2018). The water use efficiency (WUE) and Nitrogen-use efficiency (NUE) of millets are also better than popular cereals. Also, the millets are gluten-free and have a low glycemic index (Muthamilarasan and Prasad, 2021). Foxtail millet is considered an alternative sustainable protein source compared to other cereals and millets (Sachdev et al., 2021). Being a rich source of zinc and iron, the consumption of millets imparts immunity (Kumar et al., 2018). Altogether, small millets are promising cereals with the capability to ensure food security in the future. In the past 2 years, amid the recent COVID-19 pandemic, major emphasis has been shifted to the regular consumption of food that boosts immunity. Millets contain easily digestible proteins and a better essential amino acid profile than other cereal crops. These characteristics make millets nutritionally superior to other major cereals and desirable for ensuring food security amidst pandemics (Muthamilarasan and Prasad, 2021).

The genus *Setaria*, a group of panicoid grasses, belongs to the tribe Paniceae and is characterized by sterile bristles. This genus comprises more than 125 species distributed in temperate regions worldwide (Kellogg, 2017). However, foxtail millet (*S. italica*) and its wild progenitor green foxtail (*Setaria viridis*), remains the most studied millets in this genus. *S. viridis* is one of the most widely spread weeds*. Setaria italica* was domesticated from *S. viridis* ~9–11,000 years back in China near the Yellow River valley. Further, it has been widely cultivated in arid and semi-arid regions (Pant et al., 2016). Tsang et al. (2017) have reported that foxtail millet was domesticated ~5,000 years back in Taiwan. Both *S. viridis* and *S. italica* (henceforth, *S. italica* and *S. viridis* will be collectively called *Setaria*) offer several advantages as compared to the established models *viz.* rice and maize, such as short life cycle (5–6 weeks), small diploid genome (395–500 Mb), short stature (10–30 cm), C_4_ photosynthesis. Since several millet species are shown to be resilient to adverse climatic conditions and biotic and abiotic stresses (Lata et al., 2013; Muthamilarasan and Prasad, 2015), the study of the *Setaria* genus has gained popularity in the past decade. In particular, foxtail, pearl, and proso millet have been considered appealing substitutes for small grain production (Pant et al., 2016). Further, C_4_ metabolism actuates food productivity by efficiently utilizing water, carbon, and nitrogen; therefore, elucidating the C_4_ metabolic pathways is important. Considering that *Setaria* is proven valuable for C_4_ photosynthesis study, it might enable the long-term goal of engineering C_4_ traits into staple crops, rice and wheat. A rapid-cycling mini foxtail millet mutant, xiaomi, was recently presented as a model system to study the C_4_ mechanism (Yang et al., 2020).

Altogether, the physiological as well as genetic, and climate resilience attributes of *Setaria* present it as a valuable model system for research (Peng and Zhang, 2021). Although there have been considerable advances in understanding the unique traits of *Setaria*, our interpretation of the underlying mechanism of its climate resilience is still in its infancy. Recent advances in genome sequencing, RNA-seq, and the discovery of unique trait-related QTLs have further provided momentum to millet research. Analysis of the available information for *Setaria* would lead to applying current knowledge to enhance our understanding of other crop species. The present review provides a comprehensive view of genome sequencing of *Setaria*, transcriptome and proteome analysis, publicly available databases, agronomically important trait-linked markers, and characterization of genes predicted from various platforms.

## *Setaria* omics during the pre-genome sequencing era

Earlier foxtail millet was predominantly cultivated in the Chinese belts. Before the green revolution, the farmers repeatedly cultivated trait-specific landraces. Despite the consumption shift toward rice and wheat, foxtail millet was recognized for its climate resilience among marginal farmers. This led to the development of improved varieties and pure lines of foxtail millet from the “All India Coordinated Improvement Project for small millets” during the 1960s. Preceding the advent of molecular markers, the research in foxtail millet began with morphological markers. Initially, Restriction fragment length polymorphisms (RFLP) markers were used in foxtail millet to group accessions from various countries. These RFLP codominant markers detected the ribosomal DNA variability and grouped the foxtail millet genotypes into European, western European, and Asiatic lines (Schontz and Rether, 1998). The RFLP markers also successfully identified the historical recombination events with three types of length polymorphisms by atp6 probes in the mitochondrial sequences of foxtail millet (Fukunaga and Kato, 2003). Besides RFLP, the most prominent marker utilized in foxtail millet was Simple-sequence repeats (SSR). These markers were implied in varying populations to enumerate the Linkage disequilibrium (LD) values. The association analysis in *S. viridis* with SSR markers projected a higher LD decay than other cereals. Thus, the SSR markers exhibited novel marker-trait associations in foxtail millet (Wang et al., 2010). A higher diversity with a maximum LD value in various landraces in China’s yellow river detected the genetic richness in landraces than the cultivated species. Hence, these markers helped in understanding the effect of domestication and artificial selection in the cultivated lines. In this series, randomly amplified polymorphic DNA (RAPD) and Inter Simple Sequence Repeats (ISSR) markers were also utilized by Kumari et al. (2011) in analyzing the higher genetic variance in South Indian foxtail millet collections.

In addition to diversity analysis, the molecular markers also resolved the ambiguities in the phylogenetic relationship of foxtail millet. In this aspect, a set of transposon display markers were utilized in the international collection. Transposon display is a modified form of AFLP that focuses on the long terminal repeats of retrotransposons (LTRs) and miniature inverted-repeat transposable elements (MITE). These LTRs are concentrated in the centromeric and peri-centromeric regions. While MITEs are in the euchromatic regions. In foxtail millet, these LTRs and MITEs are identified in the mutant alleles of the Waxy gene (*GBSS 1*). These mutants have a TE insertion to produce a sticky endosperm and are found in the Asian collections. These TD markers identified in the waxy alleles were grouped into eight clusters by Hirano et al. (2011). In addition, 98 novel intron length polymorphic markers (ILP) from rice were used in the molecular diversity analysis. Of these, 26 ILPs successfully classified 45 accessions in *Setaria* (Gupta et al., 2011). Further, molecular diversity with 45 SSR markers having di- and tri-nucleotide repeat motifs in 12 populations of Taiwan was shown by Lin et al. (2011). The genetic maps in foxtail millet have been constructed with RFLP, SSR, and SNP markers in trisomics, RILs, and F2 populations. The RFLP-based map with 160 loci was generated from the trisomic lines in *S. italica* × *S. viridis*. The total span length of this map was 964 cM. This was the first successful attempt with 80% coverage across the chromosomes, revealing that *S. viridis* carried a gene for gamete fertility (Wang et al., 1998). After this, SSR markers were used to develop a linkage map, and their presence in the genome was predominant in the intergenic regions. With 100 SSR markers in the F2 population between *S. italica* × *S. viridis*, this map was developed with a span length of 1,645 cM. There was an uneven distribution of markers in this map, with three on chromosome 3 and 18 on chromosome 9. The distribution pattern was due to the variations in high and low copy numbers across the genome and constructed in mapmaker version 3 using the kosambi mapping function (Jia et al., 2009).

**Figure 1:**
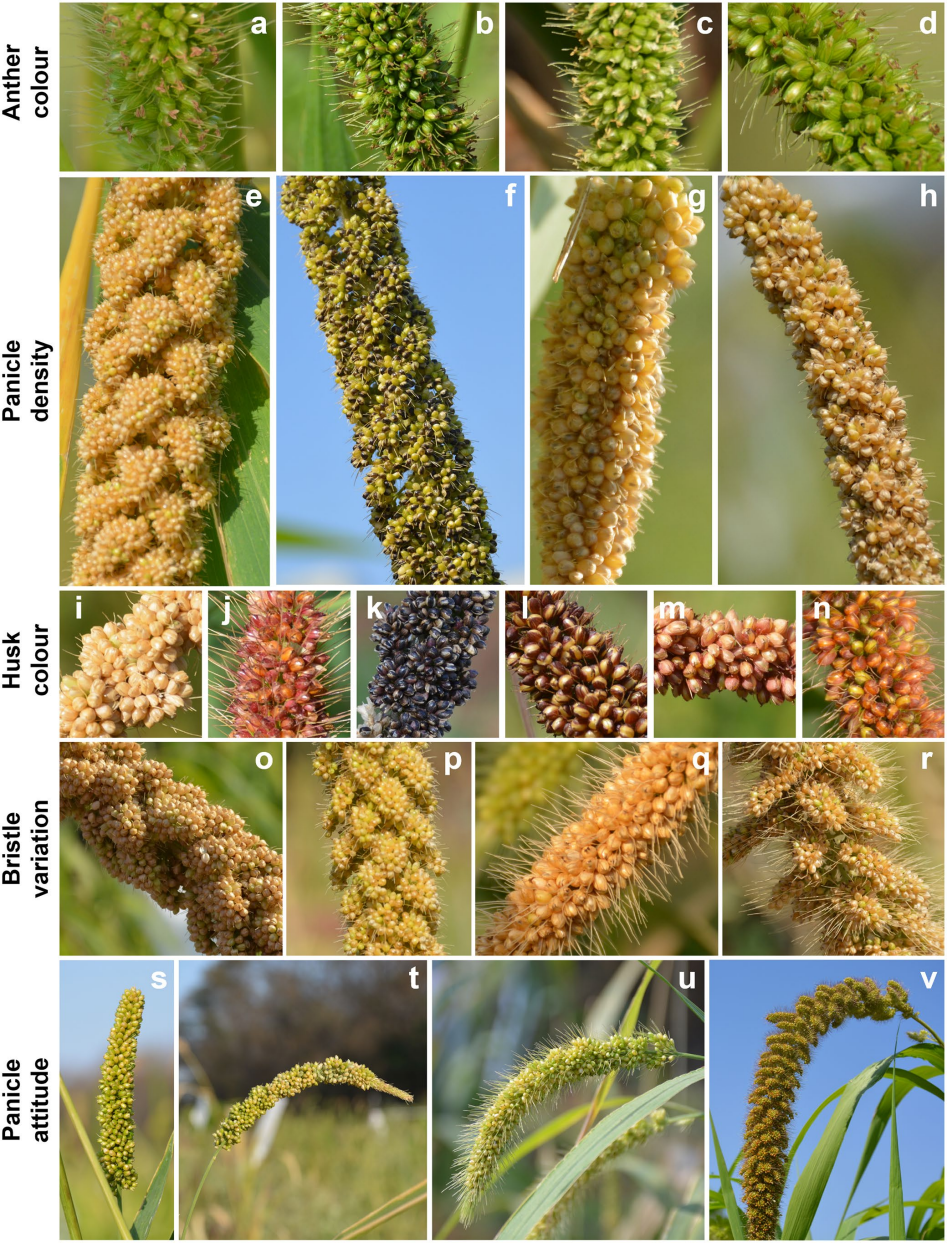
Genetic diversity of various seed-related traits in foxtail millet. Variation in anther color **(A–D)**, panicle density **(E–H)**, husk color **(I–N)**, bristles **(O–R)**, and panicle attitude **(S–V)** are shown. The names of the accessions are listed in Supplementary Table 1. figure not to scale.

The morphological traits, namely, anthocyanin pigmentation, length of bristles, plant height, non-glutinous seeds, seed size, and seed color, were used to distinguish the interspecific hybrids (Figure 1). The distribution of these traits in the F_2_ and their subsequent generations were involved in determining the nature of genes (dominance/recessive). Later, the cytological and isozyme markers were utilized in identifying their homologous pairing and the phylogeny in the related species. Using such an approach, *S. pyconoma* was found to have eight intermediate and 14 *viridis* type chromosomes, which provided information about its ancestry. Subsequently, molecular cytogenetics involving, Genomic *in situ* hybridization (GISH) and Fluorescence *in situ* hybridization (FISH) elaborated the similarities among the chromosomal structures in *S. viridis* and *S. italica*. Although the cultivated and wild species were morphologically different, they had similar cytological features. These molecular cytology tools also successfully distinguished the interspecific hybrids of *italica* x *verticillata* complex and facilitated grouping of the species in their respective gene pools (Benabdelmouna et al., 2001; Pallares et al., 2004; Brenner et al., 2015).

Further, screening of the germplasm based on their seed morphology using seed fluorescence imaging as per ISTA for varietal identification was adopted in the classical genetics’ characterization. Other essential biochemical markers utilized in the classical diversity analysis were based on the phenol test, ferrous sulfate test, and SDS-protein analysis. In the cluster analysis, the proteins quantified through SDS PAGE were implied as isozyme markers to group the accessions. Among all, the enzymes peroxidase and esterase were the crucially utilized isozymes for detecting polymorphism in foxtail millet. Thus, the pre-sequencing era in foxtail millet focused on documenting the wild species and sub-races of *S. italica*. Overall, these studies laid a foundation for understanding the allelic richness and the course of historical domestication events in the related species (Willweber-Kishiomoto, 1962; Khan, 1997; Haroun, 2002; Upadhyaya et al., 2009).

## Genome sequencing and resequencing efforts in *Setaria*

The foxtail genome was decoded by Bennetzen et al. (2012) and Zhang et al. (2012). These two findings revealed the overall genetic potential of *S. italica*. The first draft by Bennetzen et al. (2012) comprised 400 Mb across nine chromosomes with a 992-locus genetic map. The sequencing was performed by Sanger sequencing analysis in the seedlings of the cultivar Yugu 1 by Joint Genome Institute, United States. The genetic sequence revealed 40% transposable elements in which long terminal repeat retrotransposons were the most abundant, and there were around 24,000 protein-encoding genes. In addition, a genetic map based on these sequences representing 1,416 cM was constructed from the F2 of B100 × *S. viridis*. This map had 992 SNP markers identified by sequencing the 247 RIL progenies.

The second high-quality draft genome sequence of *S. italica* was reported by Zhang et al. (2012). The draft genome of 423 Mb across the nine chromosomes was sequenced by a whole-genome shotgun next-generation sequencing in the foxtail millet cultivar “Zhang gu.” The total number of genes annotated was 38,801, of which ~81% were expressed, and ~46% were transposable repeats (TE: 31.60% and DNA Transposon: 9.40%). Sequencing analysis revealed the presence of chromosome reshuffling events, where the Chr 7, Chr 9, Chr 3, and 10 of rice shared similarity with Chr 2 and Chr 9 of foxtail millet. These results represented the course of domestication events and the phylogenetic relationships of foxtail millet with rice and other cereals. In addition, A2, a photo-thermosensitive male sterile line, was also sequenced by Illumina GA II, and this was used to construct a genetic map. The comparative alignment of this sequence with Zhan gu identified 542,322 SNPs, 33,587 indels, and 10,839 structural variants (Zhang et al., 2012). Later, the draft genome sequence was utilized to tap the stress-regulated gene families in foxtail millet.

With the advancement in sequencing technologies, the resequenced genome assembly of foxtail millet was completed by Ni et al. (2017). Resequencing of 184 foxtail millet recombinant inbred lines updated the Zhang gu reference genome sequence. Approximately 16 Mb was added to the reference genome assembly as unanchored scaffolds, and the error correction with 3,158 gaps in Yugu 1 reference was completed by a bin map construction. The study further determined QTLs for nine agronomic traits with two major QTLs for plant height. These QTLs exhibited 89% similarity to the sd1 gene in rice (Ni et al., 2017). Further, QTL-seq analysis was performed involving whole-genome resequencing using pair-end sequencing with bulk segregants of F2 obtained from Shinanotsubuhime × Yuikogne for heading date (Yoshitsu et al., 2017). Genome-wide comparison of three bulks, *viz.*, early, late, and extremely late heading, revealed two associated QTLs for heading. The first QTL, qDTH2, was identified on chromosome 2 for late heading, whereas the second QTL, qDTH7, was identified on chromosome 7 for extremely late heading (Yoshitsu et al., 2017). Next, a map-based cloning and high-throughput sequencing of loose panicle 1 mutant with increased panicle lengths and grain size encoded a novel WRKY transcription factor. A single shift in the G-A transition of the fifth intron in LP1 resulted in three disorganized splicing events owing to an enlarged panicle attitude which could be explored in developing bold grains in foxtail millet` (Xiang et al., 2017).

Furthermore, the sequencing effort involving dd-RAD sequencing of 142 foxtail millet core collection identified 844 SNPs on chromosome 5–2,153 on chromosome 8 with an average SNP frequency of 25.90 per Mb. The genome-wide association study (GWAS) of this data revealed 81 trait-associated markers for 10 traits across the genome (Jaiswal et al., 2019). Recently, resequencing of 164 RILs generated from Longgu7 × Yugu 1 was also conducted to identify QTLs and SNPs. Approximately 1,047,978 SNPs were detected between the parents. Three thousand four hundred thirteen bin markers were then used to generate a map having 3,963 recombinant breakpoints. Mapping and sequencing analysis depicted 47 QTLs for four traits: straw weight, panicle weight, grain weight per plant, and 1,000-grain weight. It also detected nine stable QTL clusters mapped on chromosomes 3, 6, 7, and 9 (Liu et al., 2020b). Deep sequencing was used to develop a high-density bin map with a 3,477-marker bin, which identified 26 QTLs associated with plant height (He et al., 2021).

Owing to the innovative sequencing technologies, several cost-effective genotyping by sequencing and RNA-seq have been carried out in this era (Supplementary Table 2). Such genotyping by sequencing 328 foxtail millet landraces and 12 green foxtail accessions presented a total of 5,677 single nucleotide polymorphisms for phylogenetic construction (Hunt et al., 2021). Another deep sequencing of long non-coding RNAs (lnRNA) in foxtail millet revealed their responses toward herbicides. Two thousand five hundred forty-seven lncRNAs were identified, of which 787 were known, whereas 1760 were novel. These lncRNAs were differentially expressed across the genotypes and could be utilized in developing herbicide tolerance in foxtail millet (Wang et al., 2020).

**Figure 2:**
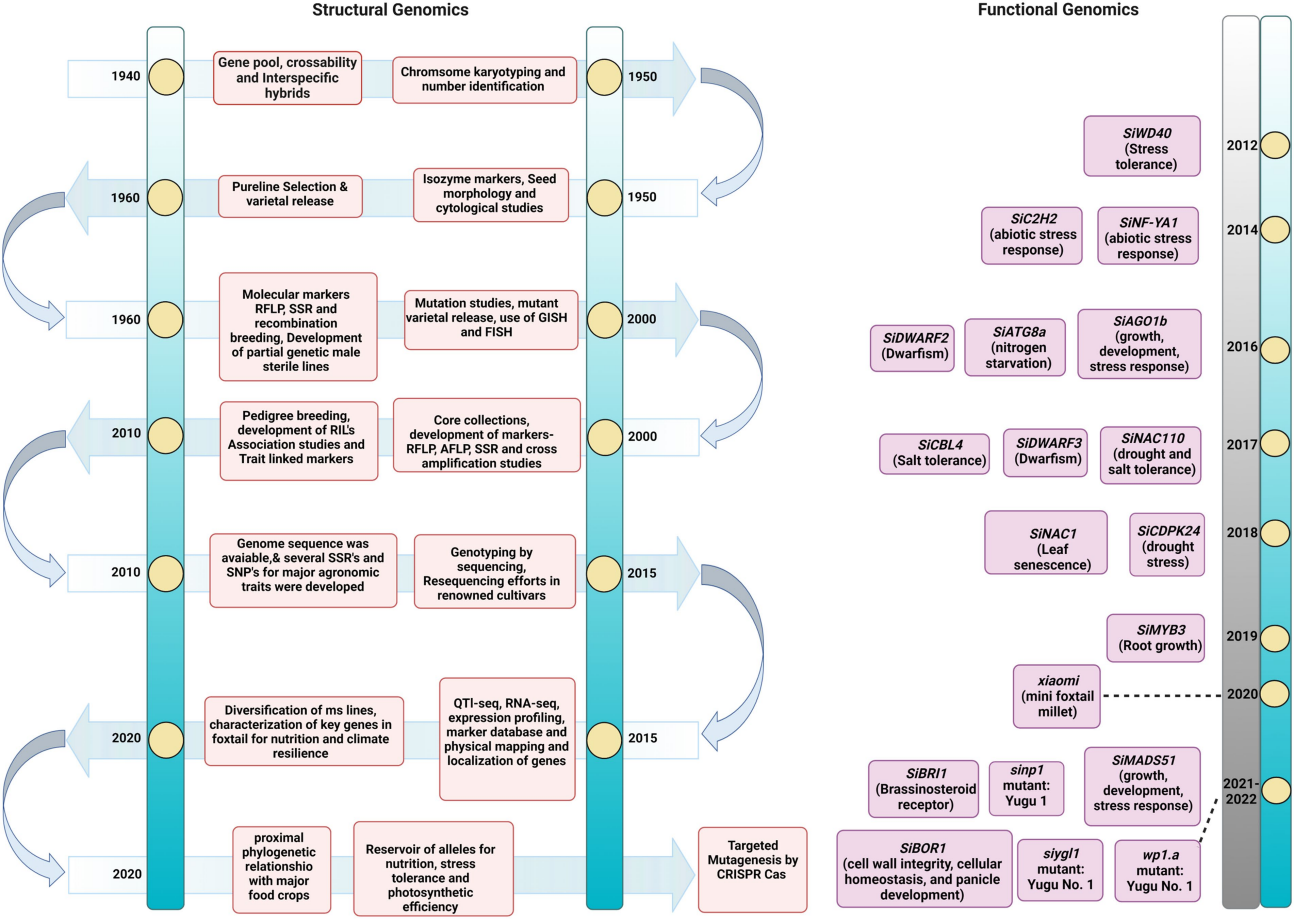
Timeline of advances in *Setaria* omics pre- and post-genome sequence release. The timeline of events in the breeding and genomics efforts completed for *Setaria*. The figure describes the significant achievements in classical and modern genetics.

Foxtail millet is known as a hardy crop. Deep resequencing of 312 local landraces was performed to dissect its adaptation mechanism to various environmental conditions. Approximately 3.02 million SNPs were detected in this genome-wide scan, and a pseudogene regulator *viz.*, SiPRR37, for heading date was identified. This was produced from a Tc1-Mariner transposon insertion in the first intron, and it was also discovered to be responsible for adaptability to harsh climates in north-eastern ecoregions (Li et al., 2021a). These novel discoveries in foxtail millet accessions enhanced the curiosity to sequence new cultivars for dissecting key traits for manipulation by breeding. In this direction, the *de novo* genome assembly of Huagu 11 compared to Yugu 1 depicted the nature of imazethapyr tolerance in foxtail millet. The assembly size was 408.37 Mb with a scaffold N50 size of 45.89 Mb. About 627 protein-encoding transcripts were identified in Huagu11 compared to 723 in Yugu 1. Here, Ser-626-Aln substitution was identified in acetohydroxy acid synthase gene to confer imazethapyr tolerance in Hugua 1. The intraspecies comparison revealed 969,596 SNPs and 156,282 indels with four chromosomal inversions (Wang et al., 2021c). The comprehensiveness and accurateness of genome sequence assemblies depend greatly on the techniques used for the task. Currently, *Arabidopsis* and rice genome sequence is the gold standard for other plant species and crops. Availability of gold standard genome sequence helps generate proper genotype and phenotype information for target gene discovery and prioritization, efficiently facilitating genome editing—however, most crops, including millets, lack gold standard genome assembly. The advancement of NGS technologies has greatly improved the closeness and correctness of genome assemblies (Kersey, 2019). In this context, the contiguity of the existing genome sequence of many crops has been improved through these NGS approaches (Belser et al., 2018). Similarly, applying advanced sequencing techniques might provide an improved genome sequence of foxtail millet, which will give a comprehensive understanding of genome architecture to discover agronomically important genes in this crop. Thus, the transition of sequencing technologies has paved the way to unravel the genomics of complex traits in foxtail millet which could be utilized in developing improved varieties (Figure 2).

## Omics resources in *Setaria*

### Genetics and genomics

Molecular markers are the key tools required for assisting the breeding programs in crops. There are studies on molecular diversity with 40 SSR markers in Taiwan landraces (Lin et al., 2012) and 77 SSR markers in green foxtail accessions (Jia et al., 2013). These programs recorded the advantageous utility of SSRs in marker-assisted breeding of foxtail millet. To facilitate the breeders of foxtail millet, a database of markers in foxtail millet was also developed by Bonthala et al. (2014). This database is a repository for genic SSRs, genomic SSRs, and ILP markers. From the reference genome sequence of Yugu 1, 5,020 highly repetitive microsatellite motifs were successfully utilized to design 788 SSR primers. These primers were analyzed in *S. italica* and *S. viridis*, resulting in successful amplicons in 733 primers. These findings were used to analyze the presence of SSRs throughout its genome (Zhang et al., 2014).

Other than SSRs, studies on developing functional miRNA-based markers from the conserved regions of foxtail millet and its related species were also developed with 100% amplification potential (Yadav et al., 2014). Later, implying ISSR markers in *Coix* and *Setaria*, their relatedness in cluster analysis was analyzed, suggesting that *Setaria* could be utilized as a model crop to tap the genetic potential of the non-sequenced crops (Dvořáková et al., 2015). After the accessibility of the whole-genome sequence in *S. italica,* the GWAS in several accessions was performed to formulate a haplotype map (Bennetzen et al., 2012; Zhang et al., 2012; Jia et al., 2013). Recent studies with SNP markers and GBS were predominantly used in association analysis. Population structure analysis among the races by Upadhyaya et al. (2015) exhibited less diversity in the indica variety. This race was comparatively unique to Moharia and maxima from the international core collection. Chander et al. (2017) incorporated 27 SSR and 4 EST-SSR in foxtail millet core collections.

Apart from analyzing the diversity for general traits, the CNL disease-resistant genes from a group of foxtail millet accessions were also used as markers in rice and Arabidopsis to identify their syntenic relationships. Among them, Si08 was similar to Os11 in rice for disease resistance (Andersen and Nepal, 2017a). Therefore, markers starting from RFLP to SNP were deployed in foxtail millet (Table 1), rendering it a model crop in cereals due to its higher transferability across species (Yadav et al., 2014). Among all the markers that were used in association analysis, it was found that the SNP markers resulted in a higher LD in germplasm. The association analysis with ddRAD approach in GWAS detected 844 SNPs in chromosome 5–2,153 SNPs in chromosome 8 (Jaiswal et al., 2019). Along with SNPs, segregation patterns and polymorphism in M2 mutants were analyzed with RAPD dominant markers (Anittha and Mullainathan, 2019). In addition, novel marker-trait analysis with ISSR markers for chlorogenic acid, catechin naringin, and myricetin concentrations presented a higher diversity for radical scavenging activity in foxtail millet, which could act as a base for stress breeding programs (Ghimire et al., 2019). The current focus with recent studies in hybrid production was also conducted to diversify the male sterile cytoplasm in foxtail millet with cpDNA markers. Four different cytoplasmic groups among the populations from cytoplasmic genetic sequences were identified, which have to be further increased in the near future to overcome cytoplasmic-specific pathogens (Liu et al., 2019a). Further, fingerprinting foxtail millet varieties using SSR and RAPD markers helped assess the genetic purity between CO (Te) 7 and the newly released variety, ATL1 (Natesan et al., 2020).

**Table 1:** List of markers developed in *Setaria* and their characteristic features.

Marker type	Characteristics	Population used	Reference
AFLP	Dominant scorable bands with PIC of 0.24 for drought	Genetic diversity for drought in 21 genotypes	Nakhoda et al. (2020)
	Dominant markers with scorable bands	14 agronomic traits in 134 genotypes for association analysis	Yazdizadeh et al. (2021)
AFLP/Transposon display markers	Variation in the insertion of TE in *GBSS* gene focuses on long terminal repeats of retrotransposons and miniature inverted repeat transposable elements	The wild and Asiatic collections for *GBSS* gene	Hirano et al. (2011)
cpDNA marker	Markers developed from chloroplast DNA from male sterile sources	Identifying male sterile cytoplasm	Liu et al. (2019a,b,c,d)
EST-SSR	Four polymorphic transferable SSRs were developed with 11 functional putative ESTs	Polymorphism in 12 cultivars	Jia et al. (2007)
	Codominant, repeatability with good generality between species	Korean landraces in *S. italica*	Ali et al. (2016)
ILP	ILP from rice, codominant with cross species amplification potential	45 accessions in *S. italica* for molecular diversity	Gupta et al. (2011)
	440 selected ILP primers with cross genera amplification were developed	4,049 ILP markers mapped to nine chromosomes	Muthamilarasan et al. (2014a)
ISSR	Co-dominant and variations between sequences	Accessions from different agro-ecological regions of India	Kumari et al. (2011)
	Co-dominant in nature with scorable and cross amplification potential	Comparative analysis in *Coix* and *Setaria*	Dvořáková et al., 2015
	Codominant markers in diversity analysis	Germplasm accessions for antioxidants like catechin	Ghimire et al. (2019)
miRNA-based marker	For identifying conserved sequences	176 pre-miRNA markers in five cultivars of *S. italica*	Yadav et al. (2014)
RAPD	Dominant and scorable markers	Accessions from different agro-ecological regions of India	Kumari et al. (2011)
	Dominant markers with scorable bands	Polymorphism in M2 mutants	Anittha and Mullainathan, 2019
RFLP	Co-dominant and length polymorphism for variation in repeats and restriction enzyme site variability	Ribosomal DNA variability in 43 accessions	Schontz and Rether (1998)
	RFLP-based linkage map with 160 loci and 964 cM	RFLP-based map in Longgu 25 × Pagoda	Wang et al. (1998)
	Co-linearity of rice and foxtail millet; transferable cDNA clones to foxtail millet	Foxtail millet-rice comparative map	Devos et al. (1998)
	Co-dominant and length polymorphism designated as type I and III based on the recombination between *atp6* genes	Length polymorphisms in mitochondrial sequences with *atp6* as probes in germplasm	Fukunaga and Kato (2003)
SNP	Trait-linked SNPs developed by deep sequencing	Accessions of *S. italica* and *S. viridis* evaluated in five locations	Jia et al. (2013)
	Co-dominant and single nucleotide polymorphisms for anthocyanin pigmentation	Land races of *S. italica* for stem color and leaf sheath	Bai et al. (2013)
	Codominant and single nucleotide polymorphisms with high quality >50% MAF	Population structure analysis in indica, moharia and maxima	Upadhyaya et al. (2015)
	Codominant and single nucleotide polymorphisms	Association analysis by ddRAD-seq based approach	Jaiswal et al. (2019)
	High-depth resequencing and single nucleotide polymorphism	*S. italica* 312 accessions	Li et al. (2021a,b)
	mGWAS-based identification of natural genetic variation in the metabolites	Metabolomics analysis of 150 millet germplasm	Wei et al. (2021)
SNP and Indels	High-Quality trait-linked markers used for map-based cloning	To identify Jingu 21 and Yugu 1 derivatives	Zhao et al. (2018)
	Identification of loci linked to the following traits: response to climate, a “loss of shattering” trait, a predictor of yield in many grass crops	Genome assembly of *S. viridis* and *de novo* assemblies for 598 wild accessions	Mamidi et al. (2020)
	Identification of SNPs and Indels and positional cloning of *Setaria* White Leaf Sheath Gene SiWLS1	*S. italica* genotype SSR41	Zhang et al. (2021a,b,c)
SSR	Codominant and variations in repeats enriched for (GA)n and (CA)n. Linkage map with 81 SSR having 1,654 cM were constructed	Polymorphic markers in F2 between B100 (*S. italica*) × A10 (*S. viridis*) were used in 40 cultivars	Jia et al. (2009)
	Codominant and variations in repeats	Association studies for detecting LD	Wang et al. (2010)
	Codominant and variations in repeats for genetic diversity	Association studies in *S. italica* landraces	Wang et al. (2012)
	Codominant and variations in di- and tri-nucleotide repeats	12 Populations in Taiwan	Lin et al. (2011)
	Codominant and variations in repeats	324 landraces of Taiwan	Lin et al. (2012)
	Codominant and variations in repeats	288 accessions in *S. viridis* for detecting LD values	Jia et al. (2013)
	Codominant and variations in repeats	159 markers mapped in *S. italica* were validated in 8 accessions	Pandey et al. (2013)
	Codominant and variations in repeats with an average PIC of 0.67	788 SSR in *S. italica* and *S. viridis*	Zhang et al. (2014)
	A high-density genetic map with SSR for QTL identification	F2 in Yugu 1 × Longgu 7 for genetic map construction	Fang et al. (2016)
	Codominant and detected 63 alleles for agronomic and nutritional traits	Genetic variability of 30 accessions in Central Himalayan region	Trivedi et al. (2018)
SSR and EST-SSR	Codominant, repeatability with a PIC of 0.31	Diversity in *S. italica* core collections	Chander et al. (2017)

A genetic map with 128 SSR markers was also successfully developed with a span length of 1239.90 cM in RILs (Qie et al., 2014). Further, the reference genome sequence of Yugu 1 was used to develop 1,013 SSR markers. These markers were implied in F2 to construct a high-density linkage map. This map was refined and produced maximum coverage of 1,035 loci across nine chromosomes. The total span length of the map was 1318.80 cM and mapped the position of 29 QTLs for 11 traits in join-map 4.3 (Fang et al., 2016). In addition to SSRs, SNP for linkage map construction in foxtail millet was used in various studies. Initially, with 992 SNP markers in RILs with a span of 1,416 cM, a linkage map was developed by Bennetzen et al. (2012). Successively, another linkage map with SNP markers in RILs identified around 59 QTLs. The total span length of the SNP formulated map was 1934.60 cM. Through multiplexed shotgun sequencing, Zhang et al. (2017a) performed a genome-wide marker discovery genotyping with MSTmap. This study also reported the location of the hybrid sterility gene on chromosome 6. Following this, next-generation sequencing methods were utilized in the reference genome sequence, which detected 2,668, 587 SNP loci across the genome. Using this data, 9,968 SNP markers in F2 were deployed to form a high-density SNP linkage map. Eleven major QTLs for eight agronomic traits were mapped on the chromosomes with MSTmap. The total span length in 9 linkage groups was 1648.80 cM (Wang et al., 2017). These constructed genetic maps could be used to identify candidate genes underlying key traits to carry out the effective marker-assisted selection in foxtail millet.

Further, map-based cloning for SiAGO1b in bulk segregation analysis and fine mapping with SSR and SNP markers refined its location in 46.30 kb region between the SNP markers SNP027326466 and SNP27372797 on chromosome 7 (Liu et al., 2016a). Following this, the positional cloning of SiYGL1 for leaf color was performed by Li et al. (2016a,b). Bulk segregation analysis in F2 for markers on chromosome 9 was focused, and it was mapped to a 288.50 kb region between the SSR markers CAAS9005 and b248. The candidate gene was refined to a 77.10 kb region between CAPS697 and InDel3595. Another approach for SisTL1 locus on chromosome 9 was performed in F2 populations with CAPS marker. This gene was refined to be located at a 91 kb interval between CAPS-8 (4,339,573 on chromosome 4) and CAPS-7 (4,430,449 on chromosome 4). The resequencing of this locus revealed three SNPs, with one in the 15th exon, the second in the intergenic region of chromosome 4 (4412036), and the third in the first exon (Tang et al., 2018). Similar studies with locus SisTL2 were fine mapped to a 12 Mb region from 46.80 Mb to 58.90 Mb on chromosome 9. This gene led to the striped leaves, and it was located in a 1 Mb region with four putative mutants. On chromosome 9, this phenotype was caused due to an alteration in G to A and found to alter a splicing site (Zhang et al., 2017a). Recent studies in *S. viridis* located the reduced leaf angle phenotype in a homozygous locus at the 800 kb region on chromosome 5. This region contained 104 disruptive SNPs and 687 indels. Also, it was found that the insertion of G at this locus Chr_5:41489494 controlled the ligule development in *S. viridis* (Mamidi et al., 2020). These gene localizations in maps could be used for determining the key factors in the expression of a phenotype in genomics-assisted breeding (Supplementary Figure 1).

Following genome sequencing and annotation, identifying genes and their functional characterization is imperative to provide researchers with tools for biological research. The summary of gene families examined in *Setaria* is given in Supplementary Table 3. A study of transcription factor families in *Setaria* led to the identification of 147 *NAC* (Puranik et al., 2013), 171 *AP2/ERF* (Lata et al., 2014), 209 *MYB* (Muthamilarasan et al., 2014b), 149 *bHLH* (Wang et al., 2018b), 124 *C2H2-ZF* (Muthamilarasan et al., 2014a), 110 *WRKY* (Muthamilarasan et al., 2015a), 44 *SCL* (Liu et al., 2017a,b), 35 *Dof* (Zhang et al., 2017b), 47 *HD-ZIP* (Chai et al., 2018) and 27 Trihelix transcription factors (Wang et al., 2018a). In addition to TFs, 53 members of SET domain-containing proteins known to catalyze histone lysine methylation were also identified (Yadav et al., 2016). Pentatricopeptide repeat (*PPR*) protein and ADP-ribosylation factors family connected to post-transcriptional processes comprised 486 and 25 members, respectively (Muthamilarasan et al., 2016; Liu et al., 2016a). Further, gene families associated with RNA silencing complex *viz.* Dicer-like (8), Argonaute (19), and RNA-dependent RNA polymerase (11) were also studied (Yadav et al., 2015). Few of these genes showed altered expression in response to abiotic stress. Another genome-wide study identified 39 Nuclear Factor Y (*NF-Y*) genes in foxtail millet. Of these, *SiNF-YA1* and *SiNF-YB8* were responsive to salt and drought stresses (Feng et al., 2015). Genome-wide analysis of the *14–3-3* family in foxtail millet and its downstream characterization revealed that Si14-3-3 proteins interact with *SiRSZ21A* (a nucleoplasmic shuttling protein) in a phosphorylation-dependent manner and are involved in multiple abiotic stress responses (Kumar et al., 2015). Analysis of different heat shock proteins, such as *HSP100, HSP90, HSP70, HSP60,* and *sHSP,* demonstrated that many of the genes were responsive during abiotic stress. In particular, expression of *SisHSP-27* was significantly increased in the foxtail millet tolerant cultivar., suggesting its importance for further characterization (Singh et al., 2016). Also, foxtail millet aquaporins *viz.* 12 plasma membrane intrinsic proteins (PIPs), 11 tonoplast intrinsic proteins (TIPs), 13 NOD26-like intrinsic proteins (NIPs), and 3 small basic intrinsic proteins (SIPs) were classified, and determination of their expression in response to various abiotic stresses highlighted the role of *SiPIP3;1* and *SiSIP1;1* in stress response (Singh et al., 2019). In addition, 11 Cytokinin oxidase/dehydrogenases linked to hormone metabolism (Wang et al., 2014) and 20 Aldehyde dehydrogenases (Zhu et al., 2014) were also identified through genome-wide analysis in *Setaria*.

*Setaria* is considered a valuable experimental system for gene discovery related to biofuel traits. In this regard, Ferreira et al. (2019) reported the analysis of gene families encoding for crucial enzymes linked to various bioenergy generation processes. Muthamilarasan et al. (2015b) described 13 gene families involved in secondary cell wall biosynthesis. In *Setaria*, 10 Phenylalanine ammonia lyase, 3 Cinnamate 4-hydroxylase, 5 4-Coumarate: CoA ligase, 2 Hydroxycinnamoyl CoA:shikimate hydroxycinnamoyl transferase, 1 p-coumarate 3-hydroxylase, 6 Caffeoyl-CoA 3-O-methyltransferase, 10 Cinnamoyl-CoA reductase, 2 Ferulate 5-hydroxylase, 6 Caffeic acid O-methyltransferase and 11 Cinnamyl alcohol dehydrogenase gene family members were investigated (Ferreira et al., 2019). Furthermore, a recent study identified 56 Laccase family members linked to lignin metabolism (Simões et al., 2020). In addition, various other gene families involved in multiple stress responses, disease resistance, seed storage or transport, such as *WD40* (Mishra et al., 2014), *14–3-3* (Kumar et al., 2015), *ZIP* (Alagarasan et al., 2017), *SOD* (Wang et al., 2018c), *OASTL* (Liu et al., 2019b), *ATG* (Li et al., 2016a,b), *LIM* (Yang et al., 2019), *CDPK* (Yu et al., 2018), *NBS-LRR* (Zhao et al., 2016), *CNL* (Andersen and Nepal, 2017b), *SSP* (Gaur et al., 2018), *MADS-box* (Zhao et al., 2021a), *BES/BZR* (Liu et al., 2021), *SNARE* (Wang et al., 2021a), and *PTI1* (Huangfu et al., 2021; Supplementary Table 3). Genome-wide identification and *in silico* analysis of phosphate transporter 1 gene (*PHT 1*) in *Setaria viridis* depicted 12 *PHT 1* gene families for phosphorous uptake (Ceasar, 2019). Karunanithi et al. (2020) identified 32 terpene synthase (TPS) gene family members in *S. italica* genome. It was also found that cytochrome P450 monooxygenase (CYP99A17) catalyzes the C19 hydroxylation of *SiTPS8* to generate the corresponding diterpene alcohols. Recently, 94 amino acid transporters (AATs) have been identified and divided into 12 subfamilies in the foxtail millet (Yang et al., 2021b). Further, characterization of the chitinase gene family in foxtail millet has identified 40 genes. Of these, a few genes might have a potential role in defense mechanisms under low temperature, drought, and osmotic stress conditions (Motukuri et al., 2021). Genome-wide identification and evolutionary analysis of the ARF gene family in foxtail millet revealed that duplication and purifying selection contribute to functional redundancy (Chen et al., 2021; Supplementary Table 3).

### Transcriptomics

**Figure 3:**
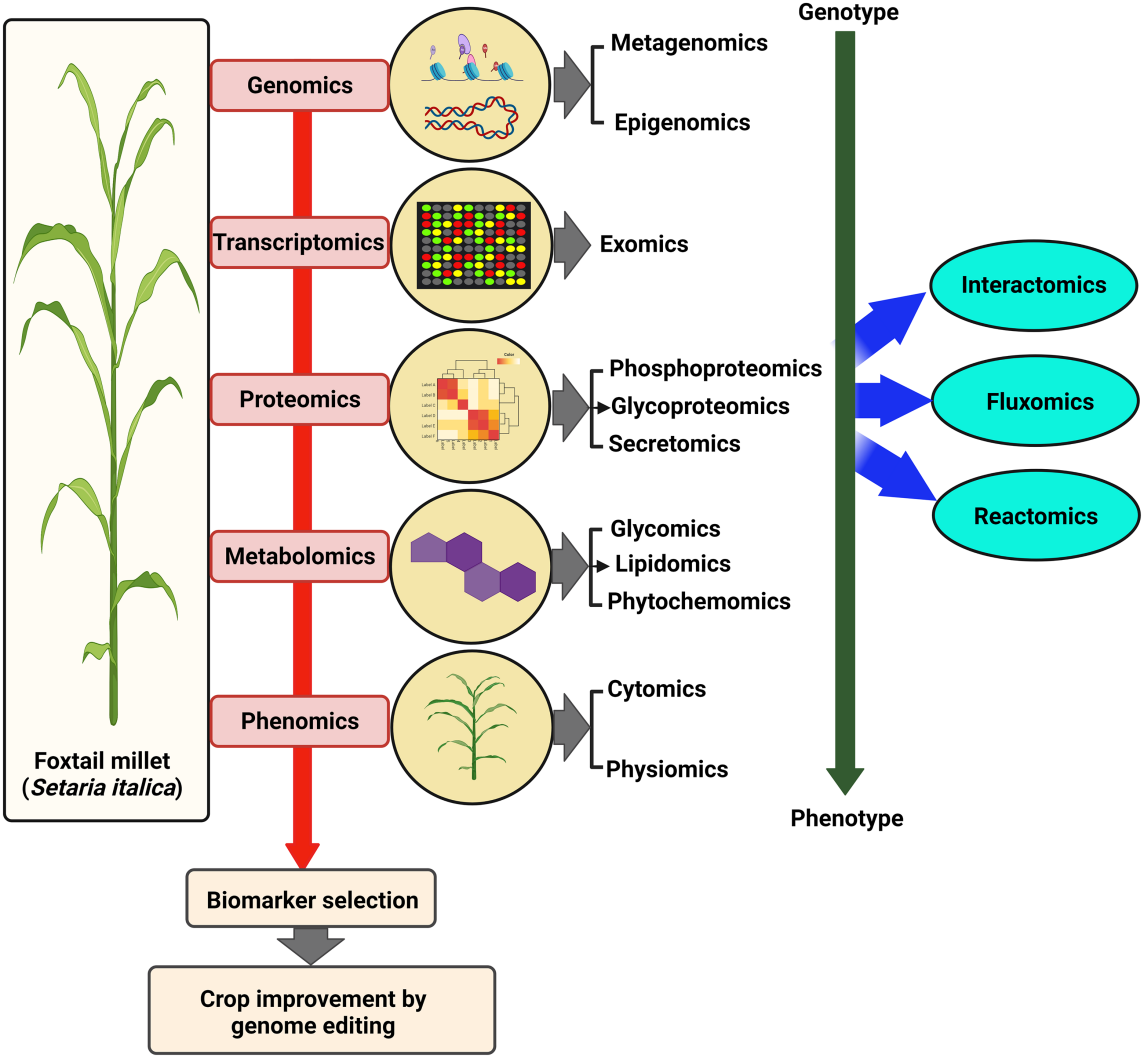
Multi-OMICS approach for crop improvement. Various advanced “OMICS” approaches used to understand different biological aspects in foxtail millet, from genotype to phenotype. These large-scale studies lead to the identification of biomarkers that paves the way for OMICS-assisted crop improvement.

Studies examining the dynamics of the whole transcriptome have hinted at the differential expression of several classes of genes playing roles during condition-dependent experimental systems (Figure 3). Earlier, suppression subtractive hybridization (SSH) based transcriptome analysis of *S. italica* led to the identification of dehydration-responsive transcripts in susceptible and tolerant cultivars (Lata et al., 2010). Another SSH-based differential gene expression analysis identified a Lipid transfer protein, *SiLTP,* that showed significant involvement in drought and salt response in foxtail millet (Pan et al., 2016). Using reference-based and *de novo* assembly, various transcripts and simple sequence repeats (SSR) involved in C_4_ photosynthesis were identified in *S. viridis* (Xu et al., 2013). In addition, transcriptome analysis of different zones of *S. viridis* internode uncovered various genes and primary metabolites involved in the transition from primary to secondary cell wall synthesis (Martin et al., 2016). Another transcriptome study of drought-tolerant *S. italica* cultivar “Yugu1” and drought-sensitive “An04” revealed the altered expression of genes involved in phytohormone metabolism and signaling, detoxification, transcription factors, and stress-related proteins (Tang et al., 2017). RNA-seq was carried out for drought-resistant M79 and its parental lines to understand the relationship between drought stress and photosynthesis. The analysis showed that photosynthetic pathway-related genes were highly expressed in M79 (Shi et al., 2018). Further, spatiotemporal transcriptome analysis of *S. viridis* inflorescence provided an overview of the regulatory network underlining sequential developmental stages (Zhu et al., 2018). Next, RNA-seq analysis of *S. italica* during water-deficit stress induced by PEG-6000 identified biological pathways involving ABA-response, proline and soluble sugar synthesis, reactive oxygen species (ROS) metabolism, channel protein genes, and transcription factors (Xu et al., 2019). Leaf transcriptome of *S. viridis* during high light and low light conditions revealed that HL and LL affect sugar accumulation and photosynthesis in a contrasting manner (Henry et al., 2020). Furthermore, differential gene expression profiling identified various genes and pathways involved in the stress response mechanism during *Uromyces setariae-italicae*, and *Sclerospora graminicola* infection in *S. italica* (Li et al., 2015, 2020).

Genome-wide gene annotation and non-coding RNA studies for drought responses by deep sequencing exhibited the role of small interfering RNAs and long non-coding RNAs. The transcriptomic sequencing explored the role of 24-nt siRNA flanking genes beneath the upregulated genes under drought stress. The PEG stimulated drought samples were initially subjected to Illumina pair-end sequencing technology. Further RNA seq analysis identified 2,824 genes responsive to drought expression patterns, and among them, 48.23% were upregulated, and 51.77% were downregulated. The key genes expressed encoded the late embryogenesis abundant protein (LEA), heat shock proteins (HSP), and aquaporin phosphatase 2C (PP2c; Qi et al., 2013). Parallel Illumina sequencing was utilized in another study to decode the role of miRNA targets toward drought stress. Degradome sequencing of the An04-4783 inbred line established 81 miRNAs belonging to 28 families. Among these, 14miRNAs were upregulated, and four were downregulated. The sequence information of the miRNAs revealed the role of 56 known genes and 26 novel, unknown genes in response to drought (Wang et al., 2016a,b).

Subsequently transcriptome analysis of mutants such as *no pollen 1*(*NP1*) characterized by defective pollen exine revealed that *SiNP1* encodes a protein involved in carbohydrate metabolism and fatty acid biosynthesis (Zhang et al., 2021a,b,c). Similarly, the characterization of *SiBOR 1* for boron accumulation in foxtail millet revealed a G-A transition at the seventh exon, and it is dominantly expressed in panicles. This mutant resulted in reduced boron with thicker cell walls in *Setaria* (Wang et al., 2021b). DNA methylation studies and transcriptome analysis revealed the role of epigenetic modifications during grain filling (Wang et al., 2021d). Recently, RNA-seq analysis of two mutants *siaux1-1* and *siaux-1-2* was performed to identify auxin-responsive factors beneath C_4_ root system architecture in shaping the root apical meristem (Tang et al., 2022). The findings pave a domain for screening the mutants to understand the C_4_ mechanism. Transcriptional profiling of foxtail millet seed at different developmental stages depicted dynamic changes in fatty acid and phytosterol content (Yuan et al., 2021).

### Proteomics and metabolomics

Proteomics and metabolomics have been employed to elucidate the protein and metabolite complement of a particular molecular network that regulates the biosynthetic, regulatory, and signaling pathways in various plant species (Figure 3). In *Setaria, the* accountability of global studies based on proteome and metabolome analyses is significantly less. Veeranagamallaiah et al. (2008) reported a 2-dimensional gel electrophoresis proteome analysis of *S. italica* during the salt stress response. Differential response of green foxtail and yellow foxtail has been studied during the application of Pyroxsulam using metabolite analysis (Satchivi et al., 2017). A proteomic study of Zhangzagu3 was performed, which is known for its climate adaptability and disease tolerance, to evaluate the parental contribution to elite traits (derived from Zhangzagu3fu and A2). This study provided valuable information related to the molecular mechanism of heterosis in hybrid millets (Song et al., 2018). Further, Tandem mass tags (TMT) followed by liquid chromatography coupled mass spectrometry (LC–MS/MS) analysis identified 321 drought-responsive proteins in foxtail millet (Pan et al., 2018). In two recent studies, changes in the proteome of foxtail millet grains during drought stress led to identifying 104 and 83 differentially abundant proteins using 2-DE (Li et al., 2019a; Xu et al., 2020). Comparative proteome profiling after the foliar application of sodium selenite has identified 123 differentially expressed proteins in foxtail millet (Liang et al., 2020). Primary and secondary metabolic profiling of PTGMS and hybrid foxtail millet lines and their comparison with rice led to identifying several species-specific metabolites accumulated during different developmental stages (Li et al., 2018). Recently, metabolome analysis of *S. viridis* roots colonized with symbiotic bacteria, Herbaspirillum seropedicae revealed that nitrogen, starch, and sucrose metabolism linked metabolites were reduced compared to the uninoculated roots. In contrast, metabolites involved in purine, zeatin, and riboflavin pathways were significantly enriched (Agtuca et al., 2020). Using iTRAQ, 610 and 276 differentially expressed proteins (DEPs) in foxtail millet, Zhangzagu10 and the female/male parent lines were identified (Weng et al., 2020).

Using a system biology-based approach, foxtail millet has also been chosen as a model to gain a deeper insight into the C_4_ photosynthetic pathway. Metabolic reconstruction, combined with transcriptome and proteome analysis, unraveled similarities and differences in the central metabolism of mature and immature tissues in *S. italica* (de Oliveira Dal’Molin et al., 2016). Integrated transcriptome and metabolome analysis explained the comprehensive regulatory network underlying drought and salinity stress response in foxtail millet (Pan et al., 2020; Yu et al., 2020). Quantitative determination of 62 volatile compounds has been performed using simultaneous distillation extraction (SDE) and gas chromatography–mass spectrometry (GC–MS) in four foxtail millet varieties, namely Jigu 42, Henggu13, Henglvgu1, and Heinuogu (Li et al., 2021b). Metabolome-based GWAS identified the basis of natural genetic variations in foxtail millet germplasm (Wei et al., 2021).

### Functional databases and online resources

After decoding the whole-genome sequence of *S. viridis* and *S. italica* (Bennetzen et al., 2012; Zhang et al., 2012), advances were made to generate large-scale genomic resources. The first-ever database for *Setaria*, the Foxtail millet Marker Database (FmMDb; http://www.nipgr.res.in/foxtail.html), was developed by Dr. Manoj Prasad Laboratory at the National Institute of Plant Genome Research (NIPGR), New Delhi, India (Bonthala et al., 2013). It provides access to genomic-, genic-SSRs, and ILP markers linking basic and applied sciences in foxtail millet. Further, the Foxtail Millet Transcription Factor Database (FmTFDb; http://59.163.192.91/FmTFDb/index.html) comprised 2,297 putative TFs belonging to 55 families (Bonthala et al., 2014). This database encompasses genomic location, sequence features, phylogeny, gene ontology (GO), and tissue-specific expression for all the TFs. In addition, the foxtail millet microRNA Database (FmMiRNADb: http://59.163.192.91/FmMiRNADb/index.html) provided marker information on 355 mature miRNAs, their secondary structure, and putative targets (Khan et al., 2014). *Setaria italica* Functional Genomics Database, SIFGD^1^ was established to predict gene function, motif analysis, regulatory modules, and gene family (You et al., 2015). This database represents genome, transcript, and protein sequence information combined with data sources like Beijing Genomics Institute, NCBI, and Phytozome. Furthermore, considering the significance of transposable elements (TE) based markers, Yadav et al. (2015) constructed the Foxtail millet Transposable Element-based Marker Database (FmTEMDb; http://59.163.192.83/ltrdb/index.html) from 30,706 TEs and 20,278 TE-based markers. In addition to the databases solely designed for *Setaria*, other public data sources, such as Plantgbd, Phytozome, and Gramene, also contain information for genome mining in millets (Duvick et al., 2008; Goodstein et al., 2012; Gupta et al., 2016).

### Functional studies on stress tolerance traits

Numerous studies have reported millets adaptation and its molecular mechanism during the abiotic stress response. Investigation of foxtail millet varieties, Zhangzagu 10 and Jingu 21 after herbicide tribenuron-methyl (TBM) treatment revealed that the grain yield decreased with increasing TBM concentration (Ning et al., 2015). In foxtail millet, high-temperature stress was linked to root-shoot growth and metabolism (Aidoo et al., 2016). Using parallel analysis of RNA ends (PARE) approach during drought stress, gene degradation and gene transcription data were combined to study uncapped mRNAs in foxtail millet (Yi et al., 2015). Gene family identification and expression analysis of *WRKY* transcription factors indicated the putative role of *SiWRKY066* and *SiWRKY082* in dehydration and salinity stress response (Muthamilarasan et al., 2015a). Drought tolerance of foxtail millet was linked to plant growth-promoting rhizobacteria (PGPR), namely, *Pseudomonas fluorescens*, *Enterobacter hormaechei*, and *Pseudomonas migulae* (Niu et al., 2018). During low-nitrogen conditions, the root system was found to be decreased compared to increased biomass in foxtail millet. Expression of transporters, SiNRT1.1, SiNRT2.1, and SiNAR2.1 in root and SiNRT1.11 and SiNRT1.12 in the shoot was also increased, resulting in enhanced nitrate uptake and remobilization (Nadeem et al., 2018). Recently, an interesting study reported that 30 mg/m3 sulfur dioxide (SO_2_) application leads to increased tolerance to drought stress by decreased stomatal apertures and a reduced leaf transpiration rate (Han et al., 2019a,b). Also, applying exogenous SO_2_ derivatives sodium sulfite and sodium bisulfite alleviates heavy metal stress in foxtail millet (Han et al., 2018). Sodium hydrogen sulfide (NaHS) treatment changes the proline content by modulating the activity of proline-5-carboxylate reductase (P5CR) and proline dehydrogenase (PDH) enzymes while combating cadmium (Cd) stress in foxtail millet (Tian et al., 2016). In another study, adding 10 mM Ca2+ to the growth medium regulated superoxide dismutase and catalase expression, thereby increasing tolerance to salt stress (Han et al., 2019a,b). Quantification of polyamines in the salt-tolerant cultivar., Prasad, and susceptible cultivar., Lepakshi revealed that increased level of polyamine during salinity stress was due to enhanced activity of spermidine synthase and S-adenosyl methionine decarboxylase enzymes in the cultivar Prasad (Sudhakar et al., 2015). Plant treatment with polyamines such as putrescine (Put) and spermidine (Spd) revealed their protective effect during salinity stress in foxtail millet (Rathinapriya et al., 2020). The HAK/KUP/KT transporter family protein in *S. italica, SiHAK1,* was overexpressed in Arabidopsis to understand the K homeostasis during K+ deficiency and salt stress which provided the clue of the potassium homeostasis mechanism in foxtail millet (Zhang et al., 2018a). Also, a study has shown the relation between an enlarged root system, increased expression of phosphate transporters *SiPHT1.1, SiPHT1.4,* and reduced expression of nitrate transporters *SiNRT2.1, SiNAR2.1* in roots during phosphate limitation (Ahmad et al., 2018).

Transcriptome analysis of low potassium stress-tolerant variety Longgu 25 led to identifying 1982 DEGs and 18 candidate genes. Further, heterologous expression of one of the candidate genes, *SiMYB3,* in Arabidopsis promoted elongation of primary roots and K^+^ deficiency tolerance (Cao et al., 2019). Overexpression of *SiMYB3* also increased low-nitrogen stress tolerance by regulating root growth in rice and Arabidopsis (Ge et al., 2019). Genome-wide identification of core ABA signaling components *SvPYL1* to *SvPYL8, SvPP2C1* to *SvPP2C12, SvSnRK2.1* to *SvSnRK2.11* and their expression profiling during salt, drought, and cold stresses have provided targets for C_4_ plant engineering (Duarte et al., 2019). Overexpression of the SET domain-containing protein *SiSET14* in yeast unfolded its role in cold stress response (Yadav et al., 2016). Liu et al. (2019c) showed the relationship between stimulating rhizosheath (layer of soil particles adhered with root surface by root hairs and mucilage) formation and declining soil water content. Recently, conserved miR394 targeted F-box gene *SiFBP6* was identified using RLM-RACE (RNA ligase mediated rapid amplification of 5′ cDNA ends), which positively regulated drought resistance in foxtail millet (Geng et al., 2021). Interestingly, P-solubilizing microbes *Acinetobacter calcoaceticus* EU- LRNA-72 and *Penicillium* sp. EU-FTF-6 has been shown to mitigate the effect of drought stress by inducing the accumulation of glycine betaine, proline, and sugars, and decreasing lipid peroxidation in foxtail millet (Kour et al., 2020). A recent study discussed various genomic designing approaches for foxtail millet for abiotic stress tolerance (Rana et al., 2021).

Among biotic stress responses, Digital gene expression (DGE) constructed from *S. italica* cultivar Shilixiang during rust pathogen *Uromyces setariae-italicae* infection shed light on the mechanism of rust-response in foxtail millet (Li et al., 2015). The transcriptional study of foxtail millet recently revealed various stress-responsive pathways during the early stage of *S. graminicola* infection (Li et al., 2020). Transcriptional profiling of foxtail millet infected with *Ustilago crameri* showed that SiCDPK and SiRboh might be positive regulators during stress response (Hao et al., 2020). In another study, two maize insect pests, namely *Spodoptera exigua* (beet armyworm; BAW) and *Spodoptera fugiperda* (fall armyworm; FAW), were found to feed on *Setaria* plants as well. The study further showed that while JA-induced response was similar in both maize and *Setaria*, secondary metabolites such as benzoxazinoids and volatiles production showed a significant difference in both the plants (Hunter et al., 2020). Foxtail millet varieties exhibit resistance against blight disease caused by *Rhizoctonia solani* Kuhn (Patro et al., 2020). An ultra-density genetic linkage map identified QTLs linked to blast resistance in foxtail millet (Tian et al., 2021). Further, the current status of blast disease in foxtail millet caused by *Pyricularia setariae* Nishikado has been shown by Das et al. (2021). Taken together, various studies have been performed elucidating the regulatory mechanisms involved in biotic and abiotic stress response in *Setaria*. A combination of OMICS-based tools can be employed in the future to decipher the key factors contributing to the stress tolerance trait in foxtail millet, drought stress in particular. This information would help researchers globally to understand the basis of stress response in plants and utilize the knowledge further to inculcate stress tolerance in other important cereal crops.

### Functional studies on biofuel traits

The Panicoideae clade comprises commercially important fuel stock grasses, such as maize, sugarcane, sorghum, and switchgrass (Lawrence and Walbot, 2007). However, the polyploid genome is one of the major limiting factors for genetic studies and manipulation of these crops. In the past decade, being genetically closer to the popular fuel stock grasses, *S. viridis* and *S. italica* are widely accepted as a promising model for biofuel research (Li and Brutnell, 2011; Muthamilarasan and Prasad, 2015; Muthamilarasan et al., 2015b; Mauro-Herrera and Doust, 2016). Petti et al. (2013) performed a deep analysis of *S. viridis* cellulose, neutral sugars, lignin, cellulose biosynthesis inhibitor response, and phylogenetic analysis of CESA genes and compared it with sorghum, maize, and switchgrass. Overexpression of *PvMYB4* in switchgrass (*Panicum virgatum*), led to increased cellulosic ethanol yield by suppressing the lignin deposition and phenolic fermentation inhibitors and balancing soluble sugars and pectic polysaccharides (Shen et al., 2013). In *S. viridis,* silencing of acyl-CoA transferase *SvBAHD01* caused enhanced biomass digestibility; therefore, it was found to be a suitable candidate for further investigation (De Souza et al., 2018). Several gene families linked to biofuel traits, namely, *PAL*, *C4H*, *4CL*, *HCT*, *C3′H*, *CCoAOMT*, *CCR*, *F5H*, *COMT*, *and CAD* have been identified in *Setaria* (Ferreira et al., 2019). Due to its small and diploid genome, *Setaria,* particularly green foxtail, appears to be a promising model to conduct future research deciphering its potential as a biofuel crop. Further, identifying regulatory pathways underlying the potential biofuel trait and an in-depth analysis of candidate genes using reverse genetics approaches have to be done to explore the genetic determinant for the biofuel attribute regulation in *Setaria*, which can be utilized further for translational research.

### Functional studies on photosynthetic traits

Fahlgren et al. (2015) developed the Bellwether Phenotyping Platform for automated recording of plant growth and multimodel phenotyping. Further, an open-source Plant Computer Vision (PlantCV) was developed for qualitative image analysis and used to detect the genotypic and environmental effect of plant height, biomass, water use efficiency (WUE), and architecture (Fahlgren et al., 2015). Various accessions have been studied to determine the WUE and heat stress tolerance in *S. viridis* (Saha et al., 2016). Thermal imaging and visible–near infrared spectroscopy were proven helpful in evaluating WUE and other physiological responses in *S. italica* (Wang et al., 2016a,b). The relationship between WUE and plant growth was also determined by Feldman et al. (2018), and the loci linked to pleiotropic components of WUE were identified through linkage mapping. Forward genetics study in *S. viridis* led to the identification of the *SvAUX1 (AUXIN1)* gene responsible for *sparse panicle1* (spp1) phenotype sharing significant homology with *ZmAUX1* in maize (Huang et al., 2017). Depletion of β-carbonic anhydrase (β-CA) in *S. viridis* indicated that photosynthesis was not affected at normal CO2 partial pressure even after the decreased level of *CA.* Also, mesophyll conductance was the limiting factor with CA only during low CO_2_ partial pressure (Osborn et al., 2017). In a similar report, CO2 assimilation was enhanced, and photosynthetic efficiency was increased during low partial pressure due to high mesophyll conductance (Ubierna et al., 2018). In *S. viridis* and *S. italica*, the correlation between photoperiod changes and flowering time was studied, indicating that flowering in *Setaria* is quicker during short-day conditions. Also, long-day conditions employ secondary genetic regulation additional to those employed by short-day photoperiod (Doust et al., 2017).

Carbon isotopic signature (δ13C) from leaf was found to be strongly linked to water use, transpiration rate, biomass, stomatal conductance, and transpiration efficiency in both *S. viridis* and *S. italica* (Ellsworth et al., 2017). Further, as an extension to this study, genetic link elucidation of this correlation identified three QTLs for δ13C_leaf_ (Ellsworth et al., 2020). In *S. italica* cultivar Yugu1 549 ethyl methane sulfonate-induced mutants were generated and screened for the disruption of Kranz anatomy, associated with C_4_ photosynthesis in many plants. About 14 mutants with abnormal Kranz structures were identified, which will help elucidate genes involved in the development of the Kranz structure (Luo et al., 2018). *SiYGL2,* the homolog of *AtEGY1*, was shown to be involved in the leaf senescence and Photosystem II function in *S. italica* (Zhang et al., 2018c). The genome-scale metabolic model was constructed in *S. viridis* that revealed the role of NH^+^_4_ and NO^−^_3_ ratio in balancing charges and 3-PGA/triosephosphate shuttle in proton balancing (Shaw and Maurice Cheung, 2019). Ermakova et al. (2019) have reported that the overexpression of Rieske FeS protein results in higher Cytochrome b6f content leading to the removal of electron transportation limit in mesophyll and bundle sheath cells. Overall, the C_4_ photosynthesis and CO_2_ assimilation level were markedly increased (Ermakova et al., 2019). Higher photosynthetic rates were also observed at elevated CO_2_ levels, and the response was analogous to C_3_ plants (Li et al., 2019b). Foxtail millet mutant library was generated, proving a valuable genetic resource for future functional studies. Further, map-based cloning identified deletion mutation in the phytoene synthase encoding WP1 exhibiting reduced chlorophyll and carotenoid in leaf and panicles (Sun et al., 2019). Transcriptome study of *S. viridis* leaves that serve as a source revealed that high light and low light exposure to a plant causes the contrasting deregulation of sugar sensors *HXK* and *SnRK1* and trehalose pathway genes (Henry et al., 2020). White light exposure increases H_2_S generation, leading to hypocotyl elongation in foxtail millet (Liu et al., 2019d). Due to C_4_ photosynthesis, and high water and nitrogen use efficiency, foxtail millet is been a focus of researchers for years. However, genetic dissection of the photosynthetic traits to establish an *in-depth* understanding of the effect of environmental factors on yield is still in infancy. Advancement in the phenomics tools has provided high-throughput, non-destructive phenotyping platforms that hold the potential to be used for studying yield-related traits more efficiently. Further, identifying and validating molecular players linked to the photosynthetic traits and WUE/NUE is imperative to reduce the environmental impact on foxtail millet production.

### Functional studies on nutritional traits

Foliar application of selenite in *S. italica* exhibited the potential of increasing Selenium and yellow pigment, thus increasing its dietary advantages (Ning et al., 2016). Numerous reports have shown the health benefits of foxtail millet, such as antiproliferative activity, lowering blood pressure, low glycemic index, glucose-lowering effect, multi-drug resistance in human HCT-8/Fu colorectal cancer, antioxidant properties, immunostimulatory activity, attenuation of Atherosclerosis (Zhang and Liu, 2015; Chen et al., 2017; Lestari et al., 2017; Ren et al., 2018a,b; Lu et al., 2018b; Marak et al., 2019; Srinivasan et al., 2020; Liu et al., 2020a). Evaluation of phenolic antioxidants of foxtail millet indicated its potential in regulating postprandial hyperglycemia (Xiang et al., 2019). Characteristics of various elements, GABA and polyphenols, β-glucan (Sharma et al., 2018b,c), and the effect of storage conditions on germination (Sharma et al., 2018a) were analyzed in *S. italica*. Gel forming ability of foxtail millet was increased significantly with the addition of CaCl_2_ and FeSO_4_, adding to its nutraceutical property (Nagaprabha and Bhattacharya, 2016). Further, physiochemical characterization of starch gel from green gram and foxtail millet was also performed by Nagaprabha et al. (2018). Terahertz time-domain spectroscopy (THz-TDS) was employed for qualitative and quantitative analyses of (a) glutamic acid and glutamine from yellow foxtail millet, and (b) ternary amino acid from foxtail millet, highlighting the application of this technique for binary and ternary amino acids measurement as compared to other methods (Lu et al., 2016, 2018a). Soluble dietary fiber content analysis from foxtail millet bran showed several health beneficial properties (Dong et al., 2019; Ji et al., 2019). Aroma compound isolation from foxtail millet grain after boiling, freeze-drying after boiling, or roasting exhibited that unsaturated aldehydes, benzene, and alcohols surged after boiling. At the same time, freeze-drying lowered the content of volatile compounds linked to the aroma. On the other hand, roasting enhanced pyrazine content (Bi et al., 2019).

In a previous study, cooked millet was also shown as promising nutraceutical food for delaying type 2 diabetes (Ren et al., 2016). Nutritional quality comparison elucidated that protein, fat, and fiber were higher in millet food products than in traditional rice products (Verma et al., 2015). *Setaria italica* resistant starch content and effect of different treatment was determined to analyze the structure and digestibility (Babu et al., 2019). In addition, the difference at the genetic level in starch physiochemical properties was determined in different accessions of Chinese foxtail millet and landraces found in Taiwan (Qi et al., 2019; Yin et al., 2019). Further, foxtail millet has been assessed for yield, quality, and morpho-nutritional traits to explore the genetic variability (Srilatha et al., 2020; Karvar et al., 2021). Sharma and Sharma (2021) showed that bioprocessing, including soaking, germination, fermentation, and a combination of aforesaid treatments, reduced the antinutrients while enhancing the bioactive profile in foxtail millet grains. Also, different milling fractions of foxtail millet were analyzed for their phenolic profiles, antioxidant properties, and α-glucosidase inhibitory effects (Zhang et al., 2021a,b,c). Further, it has been shown that the unsaturated fatty acids are responsible for forming volatiles, thus leading to an unpleasant aroma during germination in foxtail millet (Li et al., 2021b). A recent study identified 18 long non-coding RNAs (lncRNAs) in relation to grain yield and predicted them to function as miRNA target mimics (Zhao et al., 2020). The nutritional quality of foxtail millet was analyzed in response to elevated CO_2_ levels and found that *e*CO_2_ significantly enhanced the accumulation of K, Mn, Zn, and starch and promoted P accumulation. These findings advocate that foxtail millet holds great potential to provide food security and nutrition under *e*CO_2_ (Gong et al., 2021). Collectively, these studies emphasize an array of health benefits and nutritional qualities of foxtail millet. However, most of these are only limited to demonstrating the effect of genetic variability, storage, and method of cooking on nutritional qualities. Metabolomics can be employed further to illustrate the primary and secondary metabolic compounds associated with these qualities. In addition, multi-OMICs would help to understand the key players attributing to the health benefits of foxtail millet and other millets.

### Functional studies on other unique traits

*In situ* hybridization mapping revealed that the apospory-specific genomic region (ASGR) of *Pennisetum squamulatum* has collinearity with chromosome 2 of *S. italica* (Sapkota et al., 2016). The anatomical study of the abscission zone (AZ) was performed to understand the difference in AZ development in the two closely related species, *S. viridis* and its domesticate, *S. italica* (Hodge and Kellogg, 2016). Further, the anatomy of early shoot development was investigated, specifically targeting Kranz anatomy initiation (Junqueira et al., 2018). Phenotyping assays were optimized for *S. viridis* at three developmental stages, *viz.* seed germination, early seedling, and adult plant growth, which will be helpful for physiological analysis of *Setaria* species and other panicoid species grasses (Acharya et al., 2017). Carbon and nitrogen isotope ratios in different tissues (leaves and grain) were determined to implicate the necessity of accounting for it further during paleodietary reconstructions (Lightfoot et al., 2016). Interestingly, the property of the foxtail millet shell as an efficient bio-sorbent was highlighted for the absorption of Cu, Zn, Cd, and Cr ions (Peng et al., 2018). Recently, variation in the utilization of N was shown at the seedling stage in different foxtail millet varieties (Erying et al., 2020). Desai et al. (2018) established *S. viridis* as a model system to uncover the mechanism underlining the Time of Day of Anther Appearance (TAA) regulation. *In situ* localization study of phytoliths exhibited species-specific morphotypes in different tissues of *Setaria* species that could be used for taxonomic characterization of *Setaria* (Bhat et al., 2018). Root microbiome examination of foxtail millet plants sampled from two geographically isolated locations indicated that the host plant assists the growth of specific bacteria in the rhizosphere (Jin et al., 2017). Metagenomic analysis of rhizoplane and endophytic bacteria in wild *S. viridis* and domesticated *S. italica* also classified species-associated microbiome (Chaluvadi and Bennetzen, 2018). While studying the rhizome of dried foxtail millet, a gram-positive bacterial strain, *Amnibacterium setariae* was isolated and characterized (Kim et al., 2019).

Endophyte isolation from both root and panicle of *S. viridis* and *S. pumila* affirmed distinct and conserved microbial taxa across genotypes and geographical distribution (Rodríguez et al., 2018). Aihemaiti et al. (2019) studied the effect of Vanadium (V) concentration on plant growth and the uptake of essential elements. At a concentration lower than 47.4 mg/L, V positively affected the accumulation of elements, such as P, Fe, Cu, Zn, and Mo, while at a higher concentration, accumulation was reduced. Root growth was more susceptible to increasing V concentration than shoot growth in *S. viridis* (Aihemaiti et al., 2019). In foxtail millet, it was found that Iso-potentials of PEG (used for drought treatment) and laundry detergent had a more adverse effect on seed germination and overall plant growth than PEG (Heidari et al., 2019). In a recent study, iron plaque (IP) formation was observed in the less studied grass *S. parviflora* which helps plants adapt to an iron-rich environment (Oliveira de Araujo et al., 2020). Transient expression in tobacco leaves revealed that foxtail millet *PPLS1*, a bHLH transcription factor associated with *SiMYB85* controlled the purple color of pulvinus and leaf sheath (PPLS) trait used as an indicative characteristic of the authentic hybrids (Bai et al., 2020). Recently, the extract from foxtail millet leaves and the stem has shown an allelopathic effect on three different weeds (Dong et al., 2019). Interestingly, the peptides derived from foxtail millet have demonstrated antioxidant and anti-inflammatory activity in HaCaT cells and RAW264.7 murine macrophages (Ji et al., 2020).

### Breeding, molecular breeding, and speed breeding in *Setaria*

Foxtail millet is a diploid cereal free of gluten and has several nutritional emoluments. Breeding for foxtail millet to develop elite cultivars began in the 1950s (Paroda and Mal, 1989). Foxtail millet, being a self-pollinated crop with minute inflorescence, the pure-line selection was the most predominantly used method for developing superior cultivars in foxtail millet (Lata et al., 2013). This remained a barrier to exploiting the prevailing genetic richness in its gene pool. Recently, CO 6 and CO (Te) 7 were released as hybrid derivatives by overcoming these constraints by crossing using a standard hot water method of emasculation and approach method for dusting (Pramitha Lydia et al., 2021). Now, advanced molecular breeding tools are being employed to utilize this prevailing diversity in its gene pool. Several genetic diversity analyses represent the variability for all morphological, biochemical, and nutritional traits in its germplasm, proving a way to perform effective selection and hybridization techniques in foxtail millet to meet the key breeding objectives (Ghimire et al., 2019). In addition, the availability of gene-specific and trait-linked markers from association analysis is an essential tool in developing high-yielding resilient varieties in foxtail millet (Vetriventhan et al., 2014).

After the availability of a complete genome sequence (Bennetzen et al., 2012; Jia et al., 2013), the breeding of foxtail millet has accelerated to inducing targeted mutation. Foxtail millet being a diploid crop, is feasible for dissecting observable mutants. Its smaller growth duration (95 DAS) also prefers a favorable condition to raise subsequent generations to develop a homozygous line within a year. Mutation in foxtail millet began in the mid of 70s (Gupta and Yashvir, 1975) and was initially used to develop elite cultivars of the previously released version. Recently with the joined venture of omics, the mutation breeding in foxtail millet has seen a different phase of success (Mamidi et al., 2020). Anittha and Mullainathan (2019) treated CO (Te) 7 with EMS and DES in various concentrations and visualized different chlorophyll and morphological mutations in M2 generation. This study confronted that EMS was more effective in attaining desirable variants in M2 generation (Anittha and Mullainathan, 2019). Generating mutant sources in foxtail millet serves as a new source for functional genomics. Recently, a library of mutants with 1,353 independent M2 lines exhibited varying chlorophyll and morphological types developed from EMS. Of these, 16 M2 lines were resequenced, and by map-based cloning, *Wp 1* gene was identified to have a significant role in chlorophyll accumulation. The results depicted an eight-base pair deletion located at the 6th exon in LOC101786849. The wild *Wp1* gene was studied to code phytoene synthase; therefore, the *wp1* mutants exhibited reduced chlorophyll and carotenoid contents in leaves and panicles due to premature termination (Sun et al., 2019). Later, advanced approaches as a beginning of reverse genetics were initiated by analyzing *de novo* assemblies of *S. viridis*. This study identified loci for three traits, *viz.*, response to climate, non-shattering, and leaf angle, which is a major factor for yield. By incorporating CRISPR-Cas 9, the *Less shattering 1* (*SvLes 1*) was validated for its role in controlling seed shattering. Comparative analysis of this gene in *S. italica* revealed that the same gene was non-functional due to an insertion of retrotransposon on domestication (SiLes1-TE). Thus, this trait could be manipulated in cultivars to prevent shattering losses in foxtail millet.

Developing mutants by spontaneous and induced mutations also serves to study male sterility. Although several Genic Male sterility (GMS), Photoperiod Genetic Male Sterile (PGMS), and Cytoplasmic Genetic Male Sterile (CGMS) were screened in China, the successful male sterile system that is still used in hybrid seed production is Partial Genetic male sterility (PAGMS). Using this, two superior cultivars, Yugu 1 and Zhaogu 1 were developed (Diao and Jia, 2017). Recent studies on mutagenic agents also revealed that lower doses of EMS were more effective in yielding desirable mutants, whereas higher doses resulted in meiotic abnormalities (Kumar and Pandey, 2021). Hence, breeding for desirable traits by a mutation in foxtail millet requires properly handling the mutagenic agents.

The recent avenue of breeding focuses on reducing the cycle of selection in developing superior varieties, reducing the time span of releasing cultivars to the farmers. Speed breeding focuses on inducing haploids, which could be essential in achieving rapid homozygosity. Such double haploid standard and haploid induction are yet to be manifested in foxtail millet. Overcoming this, an initiative for editing of *SiMTL* gene, which is orthologous to the maize haploid inducer gene, was prompted by Cheng et al. (2021). The study successfully achieved an average haploid induction rate of 2.8%. It was also found that this could be enhanced by developing knock-out mutants of *SiMTL*. Future approaches to inducing haploids and DH techniques will be more rewarding to millet breeders in developing genetic resources that could alter the phase of foxtail millet cultivation.

### Hotspots in *Setaria* research

**Figure 4:**
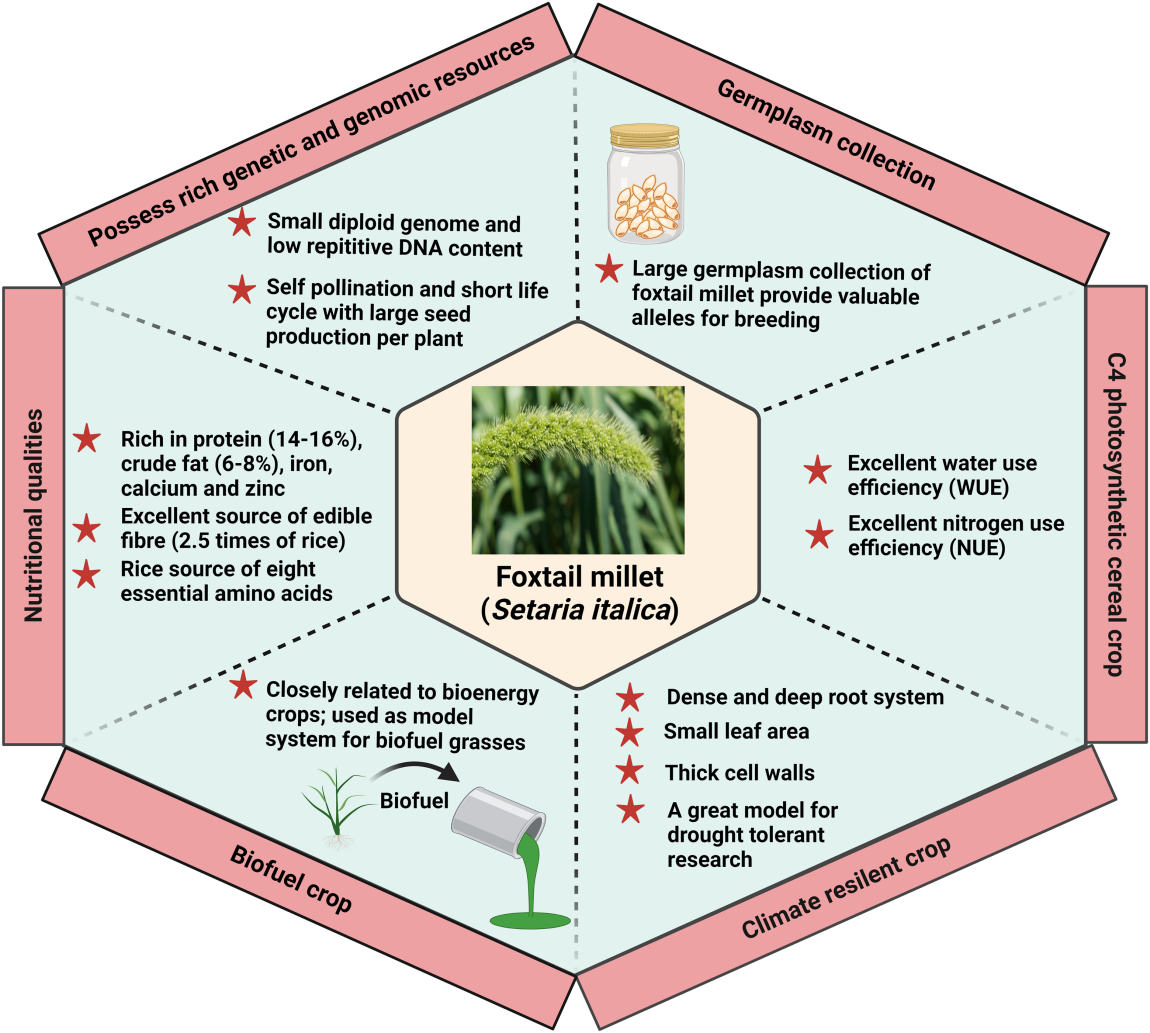
Foxtail millet as a model crop to translate the information in other crops. Foxtail millet is considered an ideal model system due to its physiological, genetic, and climate resilience attributes.

In recent years, *Setaria* has been considered an ideal model system for expanding the identification and characterization of the underlying mechanism of important agronomical traits (Figure 4). The shift in the dietary habits toward foxtail millet further focuses on improving its palatability and consumer preference for marketability. The consumer prefers a yellow-colored grain with a higher concentration of aldehydes which offer a sweet aroma. The yellow-colored grain also has a higher resistant starch content than other varieties. Molecular markers for selecting foxtail millet in rendering a preferable quality have a major thrust. Three genes control the color of seeds in foxtail millet, *viz., B*, *I,* and *K*. The genes *B* and *I* are mapped on chromosomes 7 and 9, while *K* is not mapped yet (He et al., 2015). In addition, a significant focus on reducing the amylose content to improve the cooking quality also plays a significant role in consumers’ choices. Following this, the second primary preference of the consumers is the consumption of unbroken grains in diets. Breeding for bold seeded types in foxtail millet with a maximum grain recovery during threshing is a major issue that must be resolved in the upcoming years. *Setaria* is highly nutritious and rich in protein and minerals, including iron and zinc. Inclusively, research on improving its folate and selenium content has been initiated to enrich its overall value (He et al., 2015). The Second International *Setaria* Genetics Conference, held at the Donald Danforth Plant Science Center, St. Louis, MO, United States, has highlighted the progress of research work in *Setaria* till March 2017 (Zhu et al., 2017). Research in the past decade has uncovered several aspects of *Setaria* with a major focus on the domesticated variety, *S. italica.* However, considering the importance and present demand of foxtail millet, extensive work is needed to elucidate the basis of genetic variation among their germplasm and exploit this information further for crop improvement. Precisely, identification of foxtail millet cultivars exhibiting tolerance to multiple stress needs to be conducted. Evaluation of these cultivars holds the potential of using this trait in climate-smart agriculture. In addition to the current reports presenting the transcriptome analysis of *Setaria,* proteomic and metabolomic investigation should be performed to complement the current information and finally draw a hypothesis. Further, identifying and characterizing genes involved in the biotic, abiotic, and combined stress response in *Setaria* is important to find the biomarker linked to stress tolerance.

Exploring the correlation between photosynthetic traits and different climatic conditions with the crop yield is also necessary for nutrient management and yield enhancement. High-resistant starch content is another quality that needs to be investigated in detail to understand how this can be utilized in the regular diet for health benefits and disease prevention. In a previous study, biofortification in millets was proposed as a promising approach for nutritional security (Vinoth and Ravindhran, 2017). However, due to the antinutrients such as phytic acid, tannins, and polyphenols, the bioavailability of important nutrients becomes a limiting factor. Therefore, reducing antinutrients using RNAi or genome editing tools (CRISPER, TALENs, ZFNs, etc.) and/or enhancing the bioavailability of nutrients with the targeted expression of specific promoters can be focused as the area of research to utilize the nutritional potential of foxtail millet efficiently. A major issue with millet flour is rancidity, which persists because of high-fat content and lipase activity. An effective approach should be standardized to decrease the activity of rancidity-causing enzymes, leading to improved shelf life and long-term use of millet flour. Furthermore, our understanding of the biofuel traits of *Setaria* species has to be expanded to make this trait useful while engineering other crops.

### Enhancing experimental design for the improvement of crops under ever-changing climatic conditions

Several studies have been conducted at different developmental stages or in response to multiple stresses and have identified candidate genes, which could be considered markers for crop improvement. Overexpression and knockdown of these genes have been performed in the native or heterologous system to comprehend their function. Methods for stable and efficient transformation of *Setaria* have been established for reverse genetics analysis of the genes identified from various experiments (Martins et al., 2015a,b; Saha and Blumwald, 2016; Van Eck, 2018; Rathinapriya et al., 2019; Nguyen et al., 2020; Santos et al., 2020; Sood et al., 2020). The shoot tip-based genetic transformation method has also been optimized for foxtail millet (Yang et al., 2021a). Also, the virus-mediated overexpression (VOX) vector based on Foxtail mosaic virus (genus Potexvirus) has been developed for protein expression in *Setaria* (Bouton et al., 2018). Earlier, foxtail mosaic virus (FoMV)-induced gene silencing (VIGS) was also established for functional genomics studies (Liu et al., 2016b). Further, successful attempts for gene editing and virus-induced flowering (VIF) have been made using the foxtail mosaic virus (Mei et al., 2019). Few studies have also optimized the reference gene for RT-PCR during developmental stages and/or stress responses in *Setaria* that can be used further for expression analyses (Lambret-Frotté et al., 2015; Martins et al., 2016; Nguyen et al., 2018).

Pan et al. (2014) identified and characterized a seed-specific promoter *pF128* in foxtail millet. During genome-wide analysis of the *NF-Y* gene family and following transcriptome study identified two genes (*SiNF-YA1* and *SiNF-YB8*) were highly responsive to salt and drought stresses. Overexpression of *SiNF-YA1* imparted drought and salt tolerance in Arabidopsis plants by regulating the expression of stress-related genes (Feng et al., 2015). Foxtail millet Abscisic acid stress ripening protein, SiASR1 overexpression, leads to enhanced tolerance in tobacco by modulating the expression of oxidation-related genes, *viz. NtRbohA, NtRbohB, NtCAT, NtSOD* and *NtAPX* (Feng et al., 2016). Functional characterization of *SiASR4* elucidated that ABA-responsive DRE-binding protein (*SiARDP*) was found to be upstream of the promoter region. Also, overexpression of *SiASR4* in Arabidopsis and foxtail millet imparts drought and salt tolerance *via* an ABA-dependent pathway (Li et al., 2017). Similarly, *SiLTP* identified through Suppression subtractive hybridization (SSH) analysis was also regulated by *SiARDP*. Plants overexpressing *SiLTP* showed enhanced resistance, while RNAi plants exhibited sensitivity to drought and salt stress compared to wild-type foxtail millet plants (Pan et al., 2016). *SiCDPK24* overexpression in Arabidopsis improved plant survival rate under drought stress by modulating the expression of genes, *viz. RD29A, RD29B, RD22, KIN1, COR15, COR47, LEA14, CBF3/DREB1A*, and *DREB2A* (Yu et al., 2018). Double zinc finger sequence harboring LIM domain-containing protein analysis pointed toward the presence of multiple stress-related *cis-*elements in the promoter of *SiWLIM2b*. Overexpression of *SiWLIM2b* in rice further advocated its possible role in multiple stress responses (Yang et al., 2019), making it an interesting candidate for future studies. In contrast, Kaur et al. (2018) showed the effect of overexpression of Arabidopsis *AGG3* gene in *S. viridis* that positively mediate stress response and yield. During the genome-wide study of autophagy-associated genes gene family, a novel gene *SiATG8a* was identified whose heterologous expression led to improved drought and nitrogen starvation stress in Arabidopsis and rice (Li et al., 2016a). Expressing *SiMYB3* in the heterologous system caused enhanced low-nitrogen stress tolerance by regulating root growth (Ge et al., 2019). Transgenic *S. viridis* plants overexpressing *Brachypodium distachyon MATE* gene showed increased tolerance to aluminum stress and higher root citrate exudation (Ribeiro et al., 2017). Overexpression of *SiMYB3* in Arabidopsis promoted elongation of primary roots and K^+^ deficiency tolerance (Cao et al., 2019).

Aquaporins, *SvPIP2;1* and *SvNIP2;2,* were expressed in *Xenopus laevis* oocyte to determine their water permeability. It was observed that during cell expansion in the stem, SvPIP2;1 serves as a water channel, and SvNIP2;2 mobilizes water and solutes from mature internodes (McGaughey et al., 2016). Map-based cloning identified a gene *SiYGL1* linked to yellow leaf mutation that regulates a set of genes involved in photosynthesis, thylakoid development, and chloroplast signaling in *S. italica* (Li et al., 2016b). Characterization of Argonaute mutant, *siago1b,* followed by RNA-seq based comparison of wild-type and mutant plants, highlighted *SiAGO1b* function in energy metabolism, cell growth, programmed death, and abiotic stress responses in foxtail millet (Liu et al., 2016c). Another gene involved in the developmental process, *SiTTG1,* was also found to regulate salinity and high glucose stress response in foxtail millet (Liu et al., 2017a,b). Mutation in the *Loose panicle 1* (*LP1*) gene in *S. italica* exhibited aberrant branch morphology, semi-dwarfism, and enlarged seed size (Xiang et al., 2017). Interestingly, brassinosteroid biosynthesis plays an essential role in the underlining alteration in inflorescence architecture (Yang et al., 2018). The transcription factor, *SiNAC1,* positively regulates leaf senescence in Arabidopsis in an ABA-dependent manner (Ren et al., 2018a,b). Loss of function mutation in both *SiSTL1* and *SiSTL2* genes leads to growth retardation, reduced chloroplast biogenesis, and leaf vein distances in an irregular manner in foxtail millet (Zhang et al., 2018b; Tang et al., 2019). Phosphate transporter *SiPHT1* silencing in foxtail millet promoted several lateral roots and root hair, whereas decreased total and inorganic P accumulation in root and shoot (Ceasar et al., 2017). Further, the expression of *PHT1* family genes leads to low phosphate stress tolerance in foxtail millet (Roch et al., 2020). RNAi of *SvBAHD01* causes reduced feruloylation of the cell wall in the stem, thus increasing biomass digestibility in *S. viridis* (De Souza et al., 2018). Identification and characterization of *BRASSINOSTEROID INSENSITIVE 1* (*BRI1*) showed the conserved role of *SiBR1* in BR signaling in foxtail millet (Zhao et al., 2021b).

## Conclusion

Most of the current research in *Setaria* focuses on genetic analysis-based QTL identification and transcriptome studies. However, proteome and metabolome analyses, key gene identification, and their characterization are still in infancy. *Setaria*, an annual diploid, offers scientists amenable features to explore as a model crop for tapping the hidden potential in underexplored crops. Current trends in foxtail millet also focus on its C_4_ mechanism. Identifying the regulatory genes beneath the C_4_ system establishes foxtail as a mini C_4_ model species. A collaborative effort with advanced breeding and phenotyping approaches for various agronomically essential traits is required to develop novel varieties of foxtail millet. There is also a crucial need to further explore the genetic resources of foxtail millet to identify several key molecular markers for performing trait genetics and association mapping. These findings would lead to marker-associated crop improvement in foxtail millet, and analogous markers can be studied in other related crops. Thus, applying advanced omics approaches will unravel the natural genetic variation in foxtail millet germplasm collection and open new opportunities for crop improvement in other crops. Also, foxtail millet could be used as a reference crop to understand the evolutionary relationship among other millet crops. The advancement of research and generated information helps study other panicoid grasses and related food and bioenergy crops.

## Author contributions

PA and MM conceived and outlined the review. PA, LP, and PC wrote the manuscript. PA, LP, RS, and PS prepared tables and figures. MP and MM revised the manuscript and provided additional inputs. All authors contributed to the article and approved the submitted version.

## Funding

Authors’ work in the area of millet genomics is funded by the Science and Engineering Research Board, Department of Science and Technology, Govt. of India (file no.: ECR/2017/001526), and Institute of Eminence grant (project no.: UoH-IoE-RC2-21-014) awarded to the University of Hyderabad by Ministry of Education, Govt. of India [Ref. no.: F11/9/2019-U3(A)].

## Conflict of interest

The authors declare that the research was conducted in the absence of any commercial or financial relationships that could be construed as a potential conflict of interest.

## Publisher’s note

All claims expressed in this article are solely those of the authors and do not necessarily represent those of their affiliated organizations, or those of the publisher, the editors and the reviewers. Any product that may be evaluated in this article, or claim that may be made by its manufacturer, is not guaranteed or endorsed by the publisher.

## Footnotes

^1^ http://structuralbiology.cau.edu.cn/SIFGD/

## Supplementary material

SUPPLEMENTARY FIGURE 1 Physical map showing the candidate genes identified for different traits. The physical map shows the positions of different candidate genes identified in foxtail millet. The highlighted regions in red depict their position on the respective chromosomes.
