# Supplementary material for: Multi-omics intervention in Setaria to dissect climate-resilient traits: Progress and prospects
Source: Front Plant Sci. 2022 Aug 31;13:892736. doi: 10.3389/fpls.2022.892736 (PMC9470963; doi:10.3389/fpls.2022.892736)
Supplement: Supplementary file 1 [file Table_1.doc]

**Supplementary Table 1.** Details of genetic diversity of various seed-related traits in foxtail millet as shown in Figure 1.

| **Trait** | **Label** | **Accession number** | **Characteristic feature** |
| --- | --- | --- | --- |
| **Anther colour** | a | Ise 1808 | Pink |
|  | b | Ise 914 | Black-Brown |
|  | c | Ise 525 | Yellow |
|  | d | Ise 480 | Maroon |
| **Panicle density** | e | Ise 1434 | Dense spikelets |
|  | f | Ise 1419 | moderately-loose |
|  | g | Ise 272 | less-compact |
|  | h | Ise 364 | compact |
| **Husk colour** | i | Ise 795 | Cream |
|  | j | Ise 1052 | Pink-orange |
|  | k | Ise 746 | Dark-purple |
|  | l | Ise 783 | Maroon |
|  | m | Ise 813 | Peach-pink |
|  | n | Ise 160 | Dark red-orange |
| **Bristle variation** | o | Ise 1725 | No bristle |
|  | p | Ise 1417 | Short |
|  | q | Ise 208 | Medium long |
|  | r | Ise 1511 | Long |
| **Panicle attitude** | s | Ise 472 | Erect |
|  | t | Ise 1780 | Semi-erect |
|  | u | Ise 745 | Moderately-drooping |
|  | v | Ise 1684 | Drooping |
