# Supplementary material for: Multi-omics intervention in Setaria to dissect climate-resilient traits: Progress and prospects
Source: Front Plant Sci. 2022 Aug 31;13:892736. doi: 10.3389/fpls.2022.892736 (PMC9470963; doi:10.3389/fpls.2022.892736)
Supplement: Supplementary file 3 [file Table_3.doc]

**Supplementary Table 3.** Summary of important gene families identified and characterized in *Setaria italica*

| **Gene type** | **Name of gene family** | **Notation** | **No. of genes deduced in foxtail millet genome** | **Potential candidate(s) for functional characterization (Gene ID)** | **References** |
| --- | --- | --- | --- | --- | --- |
| **Transcription factors** | NAM, ATAF and CUC | NAC | 147 | *SiNAC128* | Puranik et al. (2013) |
|  |  |  |  | *(Si036695m)* |  |
|  | APETALA2/ethylene-responsive element binding factor | AP2/ERF | 171 | *SiAP2/ERF-002* | Lata et al. (2013) |
|  |  |  |  | *(Si018222m)* |  |
|  | Myeloblastosis family | MYB | 209 | *SiMYB126* | Muthamilarasan et al. (2014a) |
|  |  |  |  | *(Si005548m)* |  |
|  | C2H2-type zinc finger | C2H2­­ ZF | 124 | *SiC2H2_031* | Muthamilarasan et al. (2014b) |
|  |  |  |  | *(Si032711m)* |  |
|  | WRKY domain-containing protein | WRKY | 110 | *SiWRKY068* | Muthamilarasan et al. (2015a) |
|  |  |  |  | *(Si013326m)* |  |
|  | SCARECROW-LIKE | SCL | 44 | *-* | Liu et al. (2017) |
|  | DNA binding with one finger | Dof | 35 | *SiDof7* and *SiDof 15* | Zhang et al. (2017) |
|  | Homeodomain leucine zipper | HD-ZIP | 47 | *Sihdz29* and *Sihdz45* | Chai et al. (2018) |
|  |  |  |  | *Seita.6G187700 and Seita.9G511500* |  |
|  | Trihelix transcription factors | TTF | 27 | *Si1g016284* | Wang et al. (2018a) |
|  | Basic helix loop helix | bHLH | 149 | *-* | Wang et al. (2018b) |
|  | BRI1-EMS suppressor/brassinazole-resistant | BES/BZR | 7 | *-* | Liu et al. (2021) |
|  | MADS-box | MADS-box | 72 | *SiMADS51* | Zhao et al. (2021a) |
|  | Auxin Response Factor | ARF | 24 | *-* | Chen et al. (2021) |
| **Histone modification** | SET | SET | 53 | *SiSET14* | Yadav et al. (2016) |
| **RNA silencing components** | Dicer-like | DCL | 8 | *SiDCL06* | Yadav et al. (2015) |
|  |  |  |  | *(Si033906m)* |  |
|  | Argonaute | AGO | 19 | *SiAGO08* |  |
|  |  |  |  | *(Si021147m)* |  |
|  | RNA-dependent RNA polymerase | RDR | 11 | *SiRDR07* |  |
|  |  |  |  | *(Si015317m)* |  |
| **Hormone metabolism** | Cytokinin oxidase/dehydrogenase | CKX | 11 | *-* | Wang et al. (2014) |
| **Aldehyde metabolism** | Aldehyde dehydrogenase | ALDH | 20 | *SiALDH2C4* | Zhu et al. (2014) |
|  |  |  |  | *(Si040073)* |  |
| **Lignin metabolism** | Laccase | LAC | 56 | *-* | Simões et al. (2020) |
|  |  |  |  |  |  |
| **Multi-process** | WD40 repeat proteins | WD40 | 225 | *SiWD40-063* | Mishra et al. (2014) |
|  |  |  |  | *(Si023326m)* |  |
|  | 14-3-3 proteins | 14.3.3 | 8 | *Si14-3-3_f* | Kumar et al. (2015) |
|  |  |  |  | *(Si010865m)* |  |
|  | ZRT and the IRT-like protein | ZIP | 36 | *Si036196, Si022298* | Alagarasan et al. (2017) |
|  |  |  |  |  |  |
|  | Superoxide dismutase | SOD | 8 | *SiCSD2* and *SiFSD3* | Wang et al. (2018c) |
|  |  |  |  | *(Seita.6G251600.1* and *Seita.9G123900.1* |  |
|  | O-acetylserine (thiol) lyase | OASTL | 10 | *SlOASTL8* and *SlOASTL9* | Liu et al. (2019b) |
|  | Autophagy-associated genes | ATG | 37 | *SiATG8a* | Li et al. (2016a) |
|  | LIM | LIM | 10 | *SiWLIM2b* | Yang et al. (2019) |
|  | Calcium-dependent protein kinases | CDPK | 29 | *SiCDPK24* | Yu et al. (2018) |
| **Secondary cell wall biosynthesis** | Cellulose synthase | CesA | 14 | *SiCesA5* | Muthamilarasan et al. (2015b) |
|  |  |  |  | *(Si028762m)* |  |
|  | Cellulose synthase-like | Csl | 39 | *-* |  |
|  | Glucan synthase-like | Gsl | 12 | *SiGsl2* |  |
|  |  |  |  | *(Si016067m)* |  |
|  | Phenylalanine ammonia lyase | PAL | 10 | *SiPAL2* |  |
|  |  |  |  | *(Si016467m)* |  |
|  | Trans-cinnamate 4-hydroxylase | C4H | 3 | *SiC4H2* |  |
|  |  |  |  | *(Si022114m)* |  |
|  | 4-Coumarate CoA ligase | 4CL | 20 | *Si4CL10* |  |
|  |  |  |  | *(Si026881m)* |  |
|  | Hydroxycinnamoyl CoA:shikimate/quinate hydroxycinnamoyl transferase | HCT | 2 | *SiHCT1* |  |
|  |  |  |  | *(Si016926m)* |  |
|  | p-Coumaroyl shikimate 3′-hydroxylase | C3H | 2 | *-* |  |
|  | Caffeoyl CoA 3-O-methyltransferase | CCoAOMT | 6 | *SiCCoAOMT3* |  |
|  |  |  |  | *(Si014344m)* |  |
|  | Ferulate 5-hydroxylase | F5H | 2 | *SiF5H2* |  |
|  |  |  |  | *(Si035174m)* |  |
|  | Caffeic acid O-methyltransferase | COMT | 4 | *SiCOMT2* |  |
|  |  |  |  | *(Si014900m)* |  |
|  | Cinnamoyl CoA reductase | CCR | 33 | *SiCCR7* |  |
|  |  |  |  | *(Si030374m)* |  |
|  | Cinnamyl alcohol dehydrogenase | CAD | 13 | *SiCAD6* |  |
|  |  |  |  | *(Si030413m)* |  |
| **Post-transcriptional process** | Pentatricopeptide repeat | PPR | 486 | *-* | Liu et al. (2016a) |
|  | ADP-ribosylation factors | ARF | 25 | *-* | Muthamilarasan et al. (2016) |
| **Disease Resistance** | Nucleotide-binding site leucine- rich repeat | NBS-LRR | 96 | *-* | Zhao et al. (2016) |
|  | Coiled-coil,Nucleotide-binding site, Leucine-rich repeat | CNL | 242 | *-* | Andersen and Nepal (2017b) |
|  | Pto-interacting 1 | PTL1 | 12 | *SiPTI1–5* | Huangfu et al. (2021) |
|  | Chitinase | CHI | 40 | *-* | Motukuri et al. (2021) |
| **Seed Storage** | Seed storage proteins | SSP | 47 | *-* | Gaur et al. (2018) |
| **Transporter** | Phosphate Transporter1 | PHT1 | 12 | *-* | Ceasar SA, (2019) |
|  | Amino acid transporter | AAT | 94 | *-* | Yang et al. (2021b) |
| **Biofuel trait** | Phenylalanine ammonia lyase | PAL | 10 | *-* | Ferreira et al. (2019) |
|  | Cinnamate 4-hydroxylase | C4H | 3 |  |  |
|  | 4-Coumarate:CoA ligase | 4CL | 5 |  |  |
|  | Hydroxycinnamoyl CoA:shikimate hydroxycinnamoyl transferase | HCT | 2 |  |  |
|  | p-coumarate 3-hydroxylase | C3′H | 1 |  |  |
|  | Caffeoyl-CoA 3-O-methyltransferase | CCoAOMT | 6 |  |  |
|  | Cinnamoyl-CoA reductase | CCR | 10 |  |  |
|  | Ferulate 5-hydroxylase | F5H | 2 |  |  |
|  | Caffeic acid O-methyltransferase | COMT | 6 |  |  |
|  | Cinnamyl alcohol dehydrogenase | CAD | 11 |  |  |
| **Aquaporin** | Plasma membrane intrinsic proteins | PIP | 12 | *SiPIP3;1 and SiSIP1;1* | Singh et al. (2019) |
|  | Tonoplast intrinsic proteins | TIP | 11 | *-* |  |
|  | NOD26-like intrinsic proteins | NIP | 13 | *-* |  |
|  | Small basic intrinsic proteins | SIP | 3 | *-* |  |
| **Stress response** | Heat shock protein 100 | HSP 100 | 20 | *SisHSP-27* | Singh et al. (2016) |
|  | Heat shock protein 90 | HSP 90 | 9 |  |  |
|  | Heat shock protein 70/DnaK | HSP 70 | 27 |  |  |
|  | Heat shock protein 60/GroE | HSP 60 | 20 |  |  |
|  | Small heat shock protein | sHSP | 37 |  |  |
|  | CBL-interacting protein kinases | CIPKs | 35 | *-* | Zhao et al. (2019) |
|  | Soluble-N-ethylmaleimide-sensitive-factor accessory-protein receptor | SNARE | 53 | *-* | Wang et al. (2021a) |
|  | Terpene synthase | TPS | 32 | *SiTPS8* | Karunanithi et al. (2020) |

- Data not available

**References**

Alagarasan, G., Dubey, M., Aswathy, K. S., and Chandel, G. (2017). Genome wide identification of orthologous ZIP genes associated with zinc and iron translocation in Setaria italica. *Front. Plant Sci*. 8, 1–11.

Andersen, E.J. and Nepal, M.P. (2017b). Data on the genome-wide identification of CNL R-genes in Setaria italica (L.) P. Beauv*. Data Br*. 13, 259–273.

Ceasar, S. A. (2019). Genome‐wide identification and in silico analysis of pht1 family genes and proteins in Setaria viridis : the best model to study nutrient transport in millets . *Plant Genome* 12, 1–9.

Chai, W., Si, W., Ji, W., Qin, Q., Zhao, M., and Jiang, H. (2018). Genome-Wide Investigation and Expression Profiling of HD-Zip Transcription Factors in Foxtail Millet (Setaria italica L.). *BioMed research international*, 2018, 8457614.

Chen, Y., Liu, B., Zhao, Y., Yu, W., and Si, W. (2021). Whole-Genome Duplication and purifying selection contributes to the functional redundancy of auxin response factor (ARF) genes in foxtail millet (Setaria italica L.). *Int. J. Genomics* 2021, 2590665.

Ferreira, S.S., Simões, M.S., Carvalho, G.G., de Lima, L.G.A., Svartman, R.M. de A., and Cesarino, I. (2019). The lignin toolbox of the model grass Setaria viridis. *Plant Mol. Biol.* 101, 235–255.

Gaur, V.S., Sood, S., Tiwari, S., and Kumar, A. (2018). Genome-wide identification and characterization of seed storage proteins (SSPs) of foxtail millet (Setaria italica (L.) P. Beauv.). *3 Biotech* 8, 1–16.

Huangfu, Y., Pan, J., Li, Z., Wang, Q., Mastouri, F., Li, Y., Yang, S., Liu, M., Dai, S., and Liu, W. (2021). Genome-wide identification of PTI1 family in Setaria italica and salinity-responsive functional analysis of SiPTI1–5. *BMC Plant Biol*. 21, 319.

Karunanithi, P.S., Berrios, D.I., Wang, S., Davis, J., Shen, T., Fiehn, O., Maloof, J.N., and Zerbe, P. (2020). The foxtail millet (Setaria italica) terpene synthase gene family. *Plant J*. 103, 781-800.

Kumar, K., Muthamilarasan, M., Bonthala, V.S., Roy, R., and Prasad, M. (2015). Unraveling 14-3-3 proteins in C4 panicoids with emphasis on model plant Setaria italica reveals phosphorylation-dependent subcellular localization of RS splicing factor. *PLoS One* 10, 1–22.

Lata, C., Gupta, S., and Prasad, M. (2013). Foxtail millet: a model crop for genetic and genomic studies in bioenergy grasses. *Crit. Rev. Biotechnol*. 33, 328–343.

Li, W., Chen, M., Wang, E., Hu, L., Hawkesford, M. J., Zhong, L., Chen, Z., Xu, Z., Li, L., Zhou, Y., Guo, C., and Ma, Y. (2016a). Genome-wide analysis of autophagy-associated genes in foxtail millet (Setaria italica L.) and characterization of the function of SiATG8a in conferring tolerance to nitrogen starvation in rice. *BMC genomics* 17, 797.

Liu, D., Cui, Y., Zhao, Z., Li, S., Liang, D., Wang, C., Feng, G., Wang, J., and Liu, Z. (2021). Genome-wide identification and characterization of the BES/BZR gene family in wheat and foxtail millet. *BMC genomics*, 22(1), 682.

Liu, D., Li, J., Lu, J., Tian, B., Liu, X., Yang, G., and Pei, Y. (2019b). Cloning and functional analysis of four O-Acetylserine (thiol) lyase family genes from foxtail millet. *Plant Physiol. Biochem*.139, 325–332.

Liu, J.M., Xu, Z.S., Lu, P.P., Li W.W., Chen, M., Guo, C.H., and Ma, Y.Z. (2016a). Genome-wide investigation and expression analyses of the pentatricopeptide repeat protein gene family in foxtail millet. *BMC Genomics* 17, 1–16.

Liu, K., Qi, S., Li, D., Jin, C., Gao, C., Duan, S., Feng, B., and Chen, M., (2017). TRANSPARENT TESTA GLABRA 1 ubiquitously regulates plant growth and development from Arabidopsis to foxtail millet (Setaria italica). *Plant Sci*. 254, 60–69.

Mishra, A.K., Muthamilarasan, M., Khan, Y., Parida, S.K., and Prasad, M. (2014). Genome-wide investigation and expression analyses of WD40 protein family in the model plant foxtail millet (Setaria italica L.). *PLoS One*. 9, e86852.

Motukuri, S.R.K., Nerella, D., Bathuru, J., Chodisetty, B., and Nallamothu, J. (2021). Genome-wide identification and in silico characterization of chitinase gene family in Foxtail millet (Setaria italica). *J. Appl. Biol. Biotechnol*. 9, 19-30.

Muthamilarasan, M., Bonthala, V. S., Khandelwal, R., Jaishankar, J., Shweta, S., Nawaz, K., and Prasad, M. (2015a). Global analysis of WRKY transcription factor superfamily in Setaria identifies potential candidates involved in abiotic stress signaling. *Front. Plant Sci*. 6, 910.

Muthamilarasan, M., Bonthala, V.S., Mishra, A.K., Khandelwal, R., Khan, Y., Roy, R., and Prasad M. (2014b). C2H2 type of zinc finger transcription factors in foxtail millet define response to abiotic stresses. *Funct. Integr. Genomics* 14, 531–543.

Muthamilarasan, M., Khan, Y., Jaishankar, J., Shweta, S., Lata, C., and Prasad, M. (2015b). Integrative analysis and expression profiling of secondary cell wall genes in C4 biofuel model Setaria italica reveals targets for lignocellulose bioengineering. *Front. Plant Sci*. 6, 1–21.

Muthamilarasan, M., Mangu, V. R., Zandkarimi, H., Prasad, M., and Baisakh, N. (2016). Structure, organization and evolution of ADP-ribosylation factors in rice and foxtail millet, and their expression in rice. *Sci. Rep*. 6, 1–13.

Muthamilarasan, M., Venkata, S. B., Pandey, G., Kumari, K, Parida, S. K., and Prasad, M. (2014a). Development of 5123 intron-length polymorphic markers for large-scale genotyping applications in foxtail millet. *DNA Res*. 21, 41–52.

Puranik, S., Sahu, P.P., Mandal, S.N., B., V.S., Parida, S.K., and Prasad, M. (2013). Comprehensive genome-wide survey, genomic constitution and expression profiling of the nac transcription factor family in foxtail millet (Setaria italica L.). *PLoS ONE* 8, e64594.

Simões, M.S., Carvalho, G.G., Ferreira, S.S., Hernandes-Lopes, J., de Setta, N., and Cesarino, I. (2020). Genome-wide characterization of the laccase gene family in Setaria viridis reveals members potentially involved in lignification. *Planta*, 251, 1–18.

Singh, R. K., Jaishankar, J., Muthamilarasan, M., Shweta, S., Dangi, A., Prasad, M. (2016). Genome-wide analysis of heat shock proteins in C 4 model, foxtail millet identifies potential candidates for crop improvement under abiotic stress. *Sci. Rep*. 6, 32641.

Singh, R.K., Shweta, S., Muthamilarasan, M., Rani, R., and Prasad, M. (2019). Study on aquaporins of Setaria italica suggests the involvement of SiPIP3;1 and SiSIP1;1 in abiotic stress response. *Funct. Integr. Genomics* 19, 587–596.

Wang, H., Hao, D., Wang, X., Zhang, H., Yang, P., Zhang, L., and Zhang, B. (2021a). Genome-wide identification and expression analysis of the SNARE genes in Foxtail millet (Setaria italica) reveals its roles in drought stress. *Plant Growth Regul*. 95, 355–369.

Wang, P., Wang, H., Wang, Y., Ren, F., and Liu, W., (2018b). Analysis of bHLH genes from foxtail millet (Setaria italica) and their potential relevance to drought stress. *PLoS ONE* 13(11): e0207344.

Wang, T., Song, H., Zhang, B., Lu, Q., Liu, Z., Zhang, S., Guo, R., Wang, C., Zhao, Z., Liu, J., and Peng, R. (2018c). Genome-wide identification, characterization, and expression analysis of superoxide dismutase (SOD) genes in foxtail millet (Setaria italica L.). *3 Biotech* 8, 486.

Wang, Y., Liu, H., and Xin, Q. (2014). Genome-wide analysis and identification of cytokinin oxidase/dehydrogenase (CKX) gene family in foxtail millet (Setaria italica). *J. Crop Prod.* 2, 244-254.

Wang, Z., Zhao, K., Pan, Y., Wang, J., Song, X., Ge, W., Yuan, M., Lei, T., Wang, L., Zhang, L., Li, Y., Liu, T., Chen, W., Meng, W., Sun, C., Cui, X., Bai, Y., and Wang, X. (2018a). Genomic, expressional, protein-protein interactional analysis of Trihelix transcription factor genes in Setaria italica and inference of their evolutionary trajectory. *BMC Genomics* 19, 1–12.

Yadav, C., Muthamilarasan, M., Dangi, A., Shweta, S., and Prasad, M. (2016). Comprehensive analysis of SET domain gene family in foxtail millet identifies the putative role of SiSET14 in abiotic stress tolerance*. Sci Rep*. 6, 32621.

Yadav, C.B., Bonthala, V.S., Muthamilarasan, M., Pandey, G., Khan, Y., and Prasad, M. (2015). Genome-wide development of transposable elements-based markers in foxtail millet and construction of an integrated database. *DNA Res*. 22, 79-90.

Yang, R., Chen, M., Sun, J. C., Yu, Y., Min, D.H., Chen, J., Xu, Z.S., Zhou, Y.B., Ma, Y.Z., and Zhang, X.H. (2019). Genome-wide analysis of LIM family genes in foxtail millet (Setaria italica L.) and characterization of the role of siwlim2b in drought tolerance. *Int. J. Mol. Sci*. 20, 1303.

Yang, Y., Chai, Y., Liu, J., Zheng, J., Zhao, Z., Amo, A., Cui, C., Quimei, L., Chen, L., and Yin-Gang Hu. (2021b). Amino acid transporter (AAT) gene family in foxtail millet (Setaria italica L.): widespread family expansion, functional differentiation, roles in quality formation and response to abiotic stresses. *BMC Genomics* 22, 519.

Yu, T.F., Zhao, W.Y., Fu, J.D., Liu, Y.W., Chen, M., Zhou, Y.B., Ma, Y.Z., Xu, Z.S., and Xi, Y.J. (2018). Genome-wide analysis of CDPK family in foxtail millet and determination of SiCDPK24 functions in drought stress. *Front. Plant Sci*. 9, 651.

Zhang, K., Fan, G., Zhang, X., Zhao, F., Wei, W., Du, G., and Zou, H. (2017). Identification of QTLs for 14 agronomically important traits in Setaria italica based on SNPs generated from high-throughput sequencing. *G3: Genes, Genomes, Genetics* 7, 1587-1594.

Zhao J, Yu A, Du Y, Wang G, Li Y, Zhao G, et al. (2019) Foxtail millet (Setaria italica (L.) P. Beauv) CIPKs are responsive to ABA and abiotic stresses. *PLoS ONE* 14(11): e0225091.

Zhao, W., Zhang, L.L., Xu, Z.S., Fu, L., Pang, H.X., Ma, Y.Z., and Min, D.H. (2021a). Genome-wide analysis of MADS-Box genes in foxtail millet (Setaria italica L.) and functional assessment of the role of SiMADS51 in the drought stress response. *Front. Plant Sci*. 12, 659474.

Zhao, Y., Weng, Q., Song, J., Ma, H., Yuan, J., Dong, Z., and Liu, Y. (2016). Bioinformatics analysis of NBS-LRR encoding resistance genes in Setaria italica. *Biochem. Genet*. 54, 232–248.

Zhu, C., Ming, C., Zhao-shi, X., Lian-cheng, L., Xue-ping, C., and You-zhi, M. (2014). characteristics and expression patterns of the Aldehyde Dehydrogenase (ALDH) gene superfamily of foxtail millet (Setaria italica L.). *PLoS ONE* 9, e101136.
