# Supplementary figures and images for: Multi-omics intervention in Setaria to dissect climate-resilient traits: Progress and prospects
Source: Front Plant Sci. 2022 Aug 31;13:892736. doi: 10.3389/fpls.2022.892736 (PMC9470963; doi:10.3389/fpls.2022.892736)

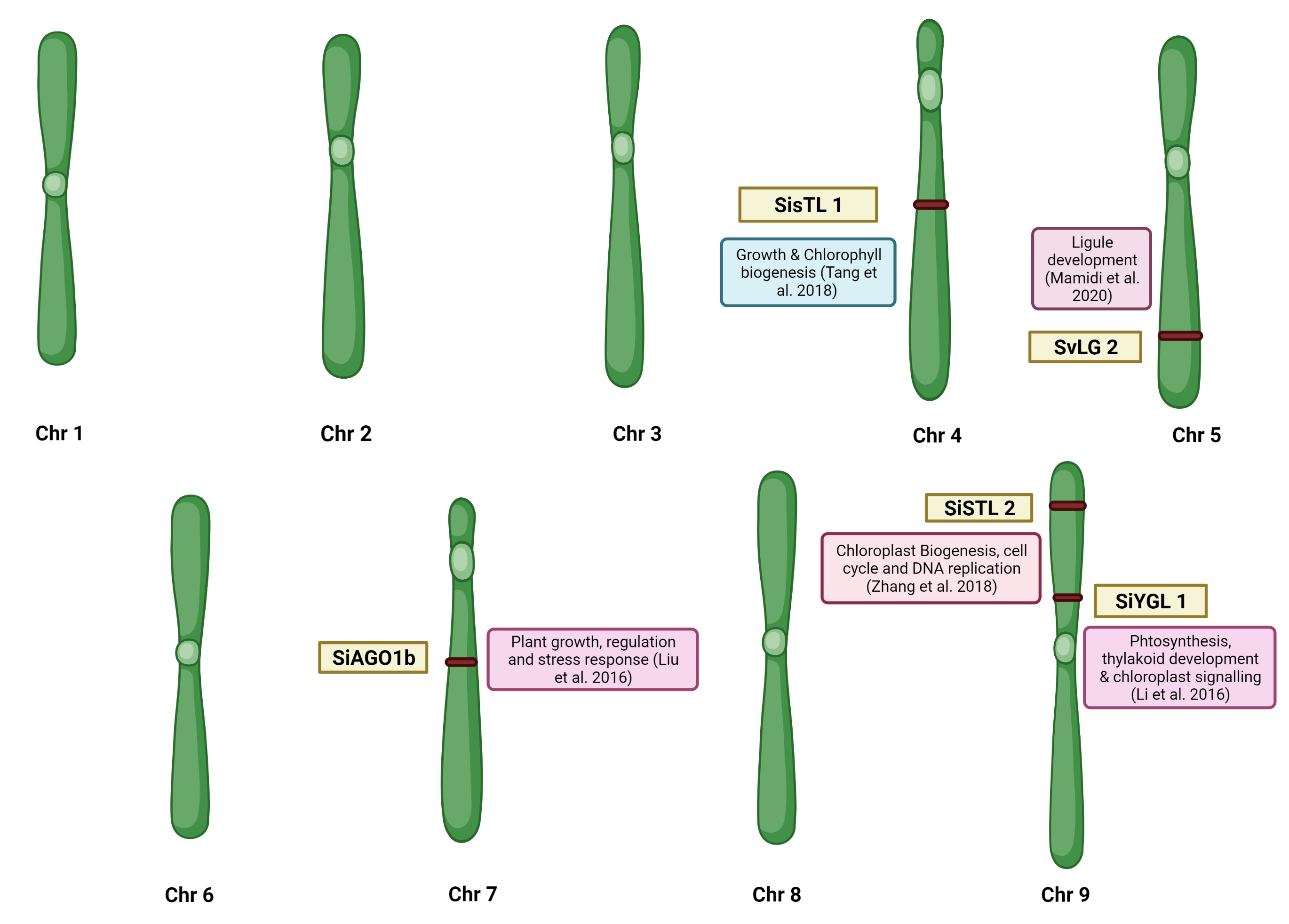

Supplement: SUPPLEMENTARY FIGURE 1 — Physical map showing the candidate genes identified for different traits. The physical map shows the positions of different candidate genes identified in foxtail millet. The highlighted regions in red depict their position on the respective chromosomes. [file Image_1.TIF]
